# Developmental plasticity of the cardiovascular system in oviparous vertebrates: effects of chronic hypoxia and interactive stressors in the context of climate change

**DOI:** 10.1242/jeb.245530

**Published:** 2024-08-07

**Authors:** Mitchell C. Lock, Daniel M. Ripley, Kerri L. M. Smith, Casey A. Mueller, Holly A. Shiels, Dane A. Crossley, Gina L. J. Galli

**Affiliations:** ^1^ Faculty of Biology, Medicine and Health, University of Manchester, Manchester M13 9NT, UK; ^2^ Division of Science, New York University Abu Dhabi, Abu Dhabi, United Arab Emirates; ^3^ Department of Biological Sciences, California State University, San Marcos, CA 92096, USA; ^4^ Department of Biological Sciences, University of North Texas, Denton, TX 76201, USA

**Keywords:** Cardiovascular, Development, Ectothermic, Hypercapnia, Hypoxia, Temperature

## Abstract

Animals at early life stages are generally more sensitive to environmental stress than adults. This is especially true of oviparous vertebrates that develop in variable environments with little or no parental care. These organisms regularly experience environmental fluctuations as part of their natural development, but climate change is increasing the frequency and intensity of these events. The developmental plasticity of oviparous vertebrates will therefore play a critical role in determining their future fitness and survival. In this Review, we discuss and compare the phenotypic consequences of chronic developmental hypoxia on the cardiovascular system of oviparous vertebrates. In particular, we focus on species-specific responses, critical windows, thresholds for responses and the interactive effects of other stressors, such as temperature and hypercapnia. Although important progress has been made, our Review identifies knowledge gaps that need to be addressed if we are to fully understand the impact of climate change on the developmental plasticity of the oviparous vertebrate cardiovascular system.

## Teaser

**Summary:** We discuss the phenotypic consequences of developmental hypoxia on the cardiovascular system of oviparous vertebrates, focusing on species-specific responses, critical windows, high-altitude adaptations and interactive effects of other stressors.

## Introduction

Oviparous (egg-laying) vertebrates typically develop in fluctuating environments with little or no parental care. This reproductive strategy has some advantages over viviparity (Shine, 2015), but it exposes the embryo to environmental stress at a critical stage of life when defence mechanisms may not be fully developed. The consequences can be severe, because environmental fluctuations during development can permanently alter organismal structure, function and behaviour, and these traits can even be inherited by subsequent generations (Sultan, 2017). Therefore, the developmental plasticity of oviparous vertebrates plays a critical role in determining their future fitness and survival. This is especially true in an era of climate change, where rising concentrations of CO_2_ in the atmosphere are driving global warming and increasing the frequency and intensity of environmental hypoxia and hypercapnia (Pörtner et al., 2014). Such rapid changes in the severity, frequency and spatial scale of these stressors will significantly challenge the developmental plasticity of oviparous species. Thus, it is important to gain an understanding of both the short- and long-term consequences of environmental stress on the embryonic physiology of these vulnerable animals.

**Glossary**

**Adrenergic**

A substance, receptor or transporter that involves adrenaline (epinephrine) or noradrenaline (norepinephrine).

**Baroreflex**

A mechanism that regulates blood pressure by altering autonomic nervous output.

**Bradycardia**

A reduction in heart rate.

**β-adrenergic sensitivity**

Sensitivity of β-adrenergic pathways to stimulation by agonists.

**Cardiac hypertrophy**

An increase in the mass or size of the heart.

**Cardiac output**

The product of heart rate (HR) and stroke volume (SV), measured in litres per minute.

**Cholinergic**

A substance, receptor or synapse that involves acetylcholine, or butyrylcholine.

**Chronic developmental hypoxia (CDH)**

Defined here as periods of hypoxia during development that last for days, weeks or months.

**Convective cardiovascular function**

The movement of solutes and O_2_ through the flow of blood.

**Critical O_2_ tension (*P*_crit_)**

The O_2_ concentration where animals transition from oxy-regulation (i.e. maintaining a stable rate of oxygen consumption as environmental oxygen concentration declines) to oxy-conformation (i.e. when oxygen consumption declines linearly with environmental oxygen concentration).

**Critical window**

Periods of heightened plasticity during development where environmental stress can affect morphology and physiology.

**Diastolic or diastole**

Referring to the stage of the cardiac cycle when the heart is relaxed.

**Eutrophication**

A process where excessive plant and algal growth occurs, mainly due to increased availability of nutrients.

**Hypercapnia**

Excess carbon dioxide.

**Hypobaric**

Having less than normal atmospheric pressure.

**Isobaric hypoxia**

Reduced O_2_ with normal atmospheric pressure.

**Oxidative phosphorylation**

A process in the mitochondria which generates ATP by the reduction of O_2_.

**Sea-level equivalent oxygen concentration**

The amount of oxygen available at high altitude that is equivalent to the oxygen concentration at sea level.

**Secretory granules**

Organelles that contain specific proteins and other macromolecules that are destined for secretion into the extracellular space.

**Systolic or systole**

Referring to the stage of the cardiac cycle when the heart is contracted.

**Tachycardia**

An increase in heart rate.

**Fig. 1.**
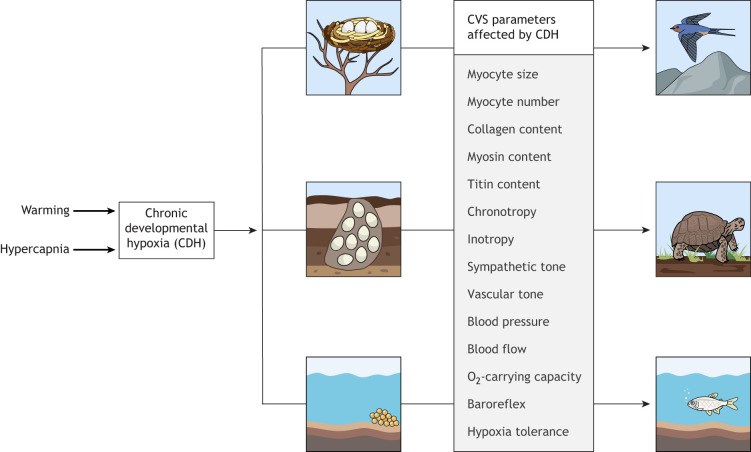
**Effects of chronic developmental hypoxia (CDH) on the cardiovascular system (CVS) of oviparous vertebrates.** CDH often develops in the nests of oviparous birds, reptiles and fish (see Box 1 for details). CDH can alter embryonic cardiovascular structure and function at multiple levels of biological organisation, and some of these abnormalities persist into adulthood (see Table S1 for full details of species-specific differences). The effects of CDH can be modulated by other environmental stressors that occur during development, including hypercapnia and warming. This figure was adapted from images created with Biorender (agreement number: NG25JUBP7L).

Oviparous vertebrates commonly experience hypoxia during embryonic development (Box 1). Importantly, studies across a wide range of species have shown that chronic developmental hypoxia (CDH; see Glossary) has persistent effects on the cardiovascular system of oviparous vertebrates (Fig. 1). It appears that some cardiovascular responses to CDH are well-conserved among mammals, birds, reptiles and fish (Galli et al., 2023; Tables S1, S2 and S3). However, there are many interspecific differences, and the outcome of CDH appears to be dependent on multiple factors, including the magnitude and duration of hypoxia, as well as developmental stage. Furthermore, the hypoxic response can be altered by the interactive effects of other environmental stressors, such as temperature and hypercapnia (Box 2). These interactions are becoming increasingly important in the context of climate change.

**Box 1. Incidence and prevalence of chronic developmental hypoxia in oviparous vertebrates**

Although most avians develop at atmospheric levels of O_2_ (∼21% saturation), megapode birds bury their eggs in mounds where O_2_ concentration can range from 13 to 17% (Seymour and Ackerman, 1980). Certain reptiles also exhibit this behaviour (mainly crocodilians and chelonians), with some nest O_2_ concentrations as low as 10% (Seymour and Ackerman, 1980). Hypoxia develops in these nests because of gas diffusion limitations, embryonic metabolism, the decomposition of matter and the activity of microorganisms (Seymour and Ackerman, 1980). Subterranean nests are also prone to flooding, which can cause unpredictable temporal changes in O_2_ (Doody and Refsnider, 2022). Many birds and reptiles also experience hypobaric hypoxia as a consequence of living at high altitude (1500 to 6500 m), where effective O_2_ concentrations can range between 10 and 19% (sea-level equivalent; León-Velarde and Monge-C, 2004). However, the most severe levels of hypoxia are observed in aquatic environments, because O_2_ concentration and diffusion rates are lower in water than in air, and they change diurnally and seasonally (Wu, 2009). For example, fish that develop in intertidal environments can transition from hyperoxia (four times air saturation) to severe hypoxia (5% O_2_ saturation) and even anoxia (zero O_2_) within 24 h (Richards, 2011). Similarly, seasonal increases in temperature can create hypoxic zones in freshwater and marine environments due to evaporation and stratification. This is particularly disruptive for sessile species that have protracted embryonic periods, such as elasmobranchs. Lastly, even in fast-flowing, well-aerated environments, embryos often experience hypoxic conditions because of low water-flow rates within the egg mass (Dhiyebi et al., 2013). These factors make fish embryos particularly vulnerable to chronic developmental hypoxia.

**Box 2. Interactions between chronic developmental hypoxia and other environmental stressors**

The phenotypic effects of chronic developmental hypoxia can be modulated by other naturally occurring or anthropogenic environmental stressors, most commonly temperature and CO_2_. In avian and reptilian nests, hypercapnia naturally occurs in parallel with hypoxia because embryonic CO_2_ production increases as the organism respires. Nest CO_2_ concentrations usually rise from ∼0.05% to 1.4%, but levels can increase to 4–12% when large amounts of decaying vegetation are present (Seymour and Ackerman, 1980). Similarly, CO_2_ fluctuations within aquatic environments can arise from natural phenomena, including variations in photosynthesis and respiration rates, wind speed and direction, ecosystem metabolism, convective mixing and ice phenology (Golub et al., 2023). All these factors are influenced by temperature, which can vary dramatically in terrestrial and aquatic developmental environments, both spatially and temporally (Du et al., 2019). Unfortunately, climate change and other anthropogenic activities are increasing the intensity of these environmental interactions. Extreme weather events, such as heat waves and flooding, are likely to increase the magnitude and duration of hypoxia and hypercapnia in terrestrial nests (Doody and Refsnider, 2022). Within aquatic environments, global warming and extreme heatwaves are increasing water temperatures in rivers (van Vliet et al., 2023), lakes (Woolway et al., 2022) and oceans (Benthuysen et al., 2020). Furthermore, the combination of eutrophication (see Glossary) and warming is increasing the prevalence and intensity of hypoxic zones. Oceanic CO_2_ levels are projected to increase from 410 to 1400 µatm by the year 2100, leading to a reduction in seawater pH of up to 0.4 units (Henson et al., 2017). Recent studies have shown that CO_2_ is also increasing in freshwater systems (Phillips et al., 2015). This problem is further confounded by anthropogenic eutrophication, which also leads to aquatic hypercapnia due to the decomposition of algal blooms (Cai et al., 2011). It is therefore critically important to study the interactive effects of hypoxia, hypercapnia and temperature on embryonic phenotypic outcomes.

The overall aim of this Review is to discuss and compare the phenotypic consequences of CDH on the cardiovascular system of oviparous birds, fish and reptiles. We define CDH here as periods of hypoxia that last for days, weeks or months. When we refer to oxygen levels, we present values as % O_2_ saturation (21% O_2_=100% air saturation). We start the Review with an overview of the effects of CDH on the embryonic cardiovascular system of each vertebrate class, as well as the persistent effects on juvenile and adult life stages. Unless specified, the data we present on juveniles and adults are taken from studies that exposed embryos to CDH for a defined period during development, and then returned them to normoxia and investigated the cardiovascular phenotype in later life. These kinds of studies reveal traits that arise from persistent developmental plasticity, rather than plasticity due to acclimation (Earhart et al., 2022). Where possible, we attempt to identify species-specific responses, the threshold for response and critical windows (see Glossary). Although data are extremely limited, we also review the interactive effects of CDH, hypercapnia and temperature on cardiovascular outcomes. Surprisingly, we were unable to find sufficient literature to warrant a review of the effects of CDH on the cardiovascular system of amphibians (only one relevant paper: Fritsche and Burggren, 1996).

## Effects of developmental hypoxia on the avian cardiovascular system

Much of what we know about the effects of CDH on the avian cardiovascular system comes from studies on domestic chickens (Table S1). These investigations were largely devised to improve farming practices or to study the clinical implications of CDH without the confounding influence of maternal responses (Itani et al., 2018). However, the levels of hypoxia used in these studies (13–17% O_2_ saturation) are within the natural range of some avian nests (Box 1), which makes them ecologically relevant.

### Effects of CDH on embryonic somatic growth and heart mass

The most common consequence of CDH is embryonic growth restriction. In chickens, isobaric or hypobaric hypoxia (see Glossary) at 13–15% O_2_ (≈2500–3500 m) consistently leads to a reduction in embryonic body mass (Table S1A), and the critical window occurs at 30–60% of incubation (Dzialowski et al., 2002; Ruijtenbeek et al., 2000). In addition, embryos from various chicken strains (broilers, red junglefowl, white Leghorn) exposed to isobaric or hypobaric hypoxia have an increased brain-to-body weight ratio (Giussani et al., 2007; Salinas et al., 2010; Skeffington et al., 2020) and/or an increased heart-to-body weight ratio (Table S1C). Asymmetric growth restriction is usually a consequence of the ‘brain-sparing’ response (Giussani, 2016), which involves a systemic vasoconstriction that shunts blood to hypoxia-sensitive organs, such as the brain and heart. Although protective in the short term, it can ultimately lead to systemic hypertension and cardiac remodelling (Giussani, 2016). Indeed, some studies have shown that isobaric or hypobaric hypoxia leads to an increase in chick embryonic heart mass, aortic wall thickness and ventricular wall thickness (Table S1B; Salinas et al., 2010; Villamor et al., 2004). However, other studies have found a decrease in heart mass, or no effect (Table S1B), and there appears to be no clear correlation between the outcome and the length or duration of the hypoxic exposure.

### Effects of CDH on embryonic O_2_-carrying capacity and cardiac function

CDH triggers a range of responses to improve O_2_-carrying capacity and delivery in vertebrates (Galli et al., 2023). Embryonic capillary density and chorioallantoic membrane (CAM) vascularity is increased in the CDH-exposed Canada goose and domestic chicken, respectively (Snyder et al., 1984; Table S1E). Haematocrit is also increased in chicken embryos exposed to CDH (13–15%, Table S1E), and the critical window lies between day 6 and 12 of development (Dzialowski et al., 2002). However, at least in the case of chickens at sea-level, the increase in embryonic O_2_-carrying capacity is not enough to offset the negative effects of hypoxia, and cardiac function is compromised. In one study, hypoxia reduced chicken embryonic ventricular peak systolic pressure, dorsal aortic peak systolic pressure (see Glossary), stroke volume and cardiac output (see Glossary), while diastolic function (see Glossary) was preserved (Sharma et al., 2006). In other studies, hypoxic chick embryos had signs of cardiomyopathy, including left ventricular dilatation, reduced ventricular wall thickness, increased apoptosis (Tintu et al., 2009), a reduced left ventricular ejection fraction, aortic thickening, reduced contractility, reduced cardiac output and diastolic dysfunction (Itani et al., 2016, 2020; Jonker et al., 2015; Rouwet et al., 2002). These problems were associated with a significant increase in cardiac oxidative stress and a reduction in cardiac antioxidant capacity (Itani et al., 2016, 2020). Heart rate is generally reduced by acute hypoxia in chickens (Akiyama et al., 1999; Altimiras and Phu, 2000; Crossley et al., 2003; Mortola et al., 2010; Sharma et al., 2006; Tazawa, 1981), but it eventually returns to control values with longer hypoxic periods; Table S1D). This is despite a significant increase in adrenal concentrations of adrenaline and noradrenaline, which is associated with a greater sensitivity of cardiac β-receptors and enhanced sympathetic innervation in the peripheral vasculature (Table S1H).

### Effects of CDH on embryonic avian cardiomyocytes

The effects of CDH on chicken embryonic cardiac structure and function are associated with multiple cellular abnormalities. Ventricular protein content and protein/DNA ratios are reduced in hypoxic embryonic chickens, which is associated with a reduction in heart mass (Asson-Batres et al., 1989). In another study, CDH initially caused cardiac myocyte hyperplasia in chicken embryos, but this eventually led to hypertrophy (see Glossary) with more myofibrils, larger Golgi complexes, less glycogen and fewer, larger secretory granules (see Glossary; Maksimov and Korostyshevskaia, 2012). This response was also accompanied by an increase in cardiac collagen (Table S1G), and a decrease in myosin heavy chain and titin proteins (Tintu et al., 2009). There is also reduced expression of genes involved in cardiac calcium handling, as well as a shift from compliant to stiff isoforms of titin and increased vascular endothelial growth factor (VEGF) expression (Jonker et al., 2015; Tintu et al., 2009). CDH also increases mitochondrial-derived oxidative stress in the hearts of chicken embryos and reduces mitochondrial efficiency and capacity (Table S1F).

### Long-term effects of avian CDH on the cardiovascular system

Numerous studies have shown that developmental hypoxia has long-term effects on the avian cardiovascular system. Firstly, the growth restriction and increased heart-to-body weight ratio associated with CDH often continues into adulthood in chickens (Table S1A; Lindgren and Altimiras, 2013). Changes in absolute heart mass are often absent after hatching or later in life, indicative of a degree of cardiac plasticity (Table S1B). However, the cardiomyopathy in hypoxic embryonic chickens observed by Tintu et al. (2009) persists into adulthood, with severe left ventricular dilatation, decreased left ventricular ejection fraction, fibrosis and diastolic dysfunction. Lindgren and Altimiras (2013) showed that adult chickens exposed to CDH have signs of systolic, but not diastolic, dysfunction and increased expression of B1 adrenoreceptors without any change in collagen content. Lastly, Skeffington et al. (2020) found a range of cardiovascular abnormalities in adult chickens exposed to CDH, including hypertension, increased cardiac work, enhanced baroreflex gain (see Glossary), left ventricular wall thickening and increased contractility. Overall, adult chickens exposed to CDH share many of the pathological cardiovascular signatures observed in adult mammals from hypoxic pregnancies (Table S3; Itani et al., 2018), and the phenotype is reminiscent of both compensated and decompensated heart failure. This makes chickens an excellent model for studying the programming of cardiovascular disease by CDH in the absence of confounding maternal factors.

## Effects of developmental hypoxia on the cardiovascular system of reptiles

Many embryonic reptiles experience CDH as part of their natural development, but climate change is increasing the frequency and intensity of these events (Boxes 1 and 2). It is particularly interesting to study cardiovascular programming in reptiles, because cardiac design differs substantially between the reptilian classes (Burggren et al., 2020). Most turtles (testudines), snakes and lizards (squamates) have a single undivided ventricle with no pressure separation between the pulmonary and systemic circulations. However, monitor lizards and pythons have a functionally divided ventricle, and crocodilians (alligators, crocodiles, caimans and gharial) have a fully divided ventricle, allowing for high systemic arterial pressures and an elevated metabolic rate. These differences place variable metabolic demands on the reptilian cardiovascular system, which could be expected to lead to species-specific responses to CDH.

### Effects of CDH on reptilian embryonic somatic growth and heart mass

As in chickens, there is no effect of CDH on reptilian embryonic body mass if the O_2_ concentration is at or above 17% (Table S1A). However, isobaric and hypobaric hypoxia at O_2_ concentrations of 10–15% consistently reduces embryonic body mass and/or body length in American alligators, snapping turtles, Florida red-bellied turtles, common wall lizards, viperine snakes and leopard geckos; but total incubation time is unchanged (Table S1A). The critical window for growth restriction is between 70 and 90% incubation in American alligators (Tate et al., 2016), whereas embryonic mass in common snapping turtles is dependent on the total hypoxic exposure time (Tate et al., 2015). Hypoxia also causes an increase in the total amount of yolk present at the end of development in American alligators, Florida red-bellied turtles, common wall lizards and viperine snakes; indicating a reduced conversion of yolk to tissue (Crossley et al., 2017; Crossley and Altimiras, 2005; Kam, 1993; Owerkowicz et al., 2009).

CDH is also associated with an increase in heart-to-body weight ratio in American alligators, snapping turtles and Florida red-bellied turtles (Table S1C). The critical window for the response in American alligators is at 20–40% of development (Tate et al., 2016), and 50–70% in common snapping turtles (Tate et al., 2015). The asymmetric growth restriction suggests that reptiles exhibit the brain-sparing response, which is supported by a recent study that found a modest increase in brain blood flow in embryonic turtles exposed to CDH (Sartori et al., 2019). An increase in absolute heart mass is also evident in hypoxic embryonic snapping turtles, lizard geckos and common wall lizards (Table S1B). However, in most studies, absolute heart mass does not change with hypoxia, suggesting that heart growth is preserved at the expense of somatic growth. Nevertheless, Crossley's laboratory thoroughly investigated the critical windows for this response and showed that cardiac enlargement occurs before somatic growth restriction (Tate et al., 2015, 2016). This finding suggests that cardiac enlargement in reptiles is a direct response to CDH, rather than a consequence of reduced somatic growth.

### Effects of CDH on reptilian embryonic O_2_-carrying capacity and heart function

In contrast to birds, CDH leads to long-term changes in reptilian heart rate, but the responses are species-specific. CDH causes bradycardia in American alligator embryos (70–90% development, 10% O_2_) and common wall lizards chronically exposed to high-altitude hypoxia [15–17% O_2_ sea-level equivalent (SLE, see Glossary); Table S1D], but it causes a significant tachycardia (see Glossary) in embryonic snapping turtles (10% O_2_) and scincid lizards (Table S1D). The underlying reason for these species-specific differences is unknown, and it is also unclear why reptiles modulate heart rate during CDH, whereas mammals and birds do not (Table S1D; Table S2C).

As in birds, chronic levels of hypoxia in embryonic reptiles trigger adaptive cardiovascular responses that improve O_2_-carrying capacity and delivery. American alligators and Florida red-bellied turtles increase haematocrit during chronic hypoxia exposure (Kam, 1993; Warburton et al., 1995), but haemoglobin isoform expression and affinity is unchanged (Bautista et al., 2021; Grigg et al., 1993). CDH also increases angiogenesis in the CAM in American alligators (Corona and Warburton, 2000), which lowers the resistance of the chorioallantoic circulation by adding parallel vascular beds. This response ultimately reduces systemic blood pressure (Crossley and Altimiras, 2005; Eme et al., 2011b, 2013). The critical window for hypotension is at 20–70% of development in snapping turtles and 50–70% in American alligators (Tate et al., 2015, 2016). However, despite arterial hypotension, blood flow to the American alligator CAM increases during hypoxia, which presumably serves to enhance gas exchange (Eme et al., 2011a; Sartori et al., 2019). Given that total blood flow remains constant, the increase in CAM blood flow may be driven by increased intraembryonic vascular resistance, which could also explain the observed cardiac enlargement in snapping turtles and lizard geckos (Eme et al., 2021; Parker and Dimkovikj, 2019).

### Effects of CDH on the embryonic reptilian acute hypoxia tolerance

In addition to baseline changes in cardiovascular function, CDH alters the embryonic cardiovascular response to acute hypoxia in reptiles. American alligator and snapping turtle embryos exposed to CDH have an attenuated response to an acute hypoxic challenge, with blunted heart rate and blood pressure responses (Crossley and Altimiras, 2005; Eme et al., 2011b). In agreement with these findings, critical O_2_ tension (*P*_Crit_; see Glossary) is lower in snapping turtles and American alligator embryos exposed to CDH, compared with their normoxic counterparts (Crossley et al., 2017; Kam, 1993). However, the enhanced hypoxia tolerance does not appear to be associated with mitochondrial remodelling (Galli et al., 2016). Collectively, these results suggest that embryos exposed to CDH are less responsive to acute hypoxic stress and may tolerate lower levels of hypoxia.

### Long-term effects of CDH on the reptilian cardiovascular system

Most of our understanding of the long-term effects of CDH have come from studies on American alligators and common snapping turtles. It is interesting to compare and contrast these two reptiles because crocodilians are archosaurs and more closely related to birds than testudines and squamates (Brusatte et al., 2010). Given that crocodilians also have a fully divided heart and higher metabolic rates, one may expect American alligators to respond to CDH more similarly to birds than to snapping turtles.

Juvenile American alligators and snapping turtles exposed to CDH most commonly experience catch-up growth, but some studies have reported persistent growth restriction (Table S1A), as well as an increased heart-to-body weight ratio (Crossley et al., 2022; Galli et al., 2016, 2021; Joyce et al., 2018; Ruhr et al., 2021; Smith et al., 2023, 2019). Despite cardiac enlargement, most resting cardiovascular parameters in juvenile American alligators and snapping turtles are similar between individuals from normoxic or hypoxic incubations. In particular, the systemic and pulmonary hypertension, as well as systolic and diastolic ventricular dysfunction that is often present in mammals and birds exposed to CDH appears to be absent in American alligators and turtles (Table S3). However, there are some reptilian cardiovascular parameters that are permanently affected by CDH. Left ventricular stroke volume is increased and pulmonary blood flow is decreased in juvenile American alligators exposed to CDH (Joyce et al., 2018; Smith et al., 2019). Likewise, heart rate is reduced, and total cardiac output is increased in juvenile common snapping turtles exposed to CDH (Wearing et al., 2017, 2016).

More differences in the long-term cardiovascular phenotype are revealed when reptiles are placed under physiological stress. Compared to normoxic controls, juvenile American alligators from hypoxic incubations that are swimming or stimulated with β-adrenergic agonists (see Glossary) have a faster rate of ventricular relaxation, greater left ventricle stroke volume, increased carotid blood flow and lower pulmonary blood flow (Joyce et al., 2018; Smith et al., 2019). Furthermore, the blunted cardiovascular response to acute hypoxia that is observed at the embryonic level is also present in juvenile alligators, suggesting a long-term improvement in hypoxia tolerance (Crossley et al., 2022, 2023; Smith et al., 2019). This is also the case for juvenile turtles exposed to CDH, as they are able to maintain cardiac output two-fold higher than controls during 2 h of anoxia (Ruhr et al., 2021). The improved anoxia tolerance is also apparent at the cellular level, and is associated with increased myofilament calcium sensitivity, a superior ability to suppress cardiac myocyte reactive oxygen species (ROS) production during anoxia and lower basal cardiac ROS production (Galli et al., 2021; Ruhr et al., 2019). These adaptations could be useful for turtles in juvenile and adult life stages, as they often experience long bouts of anoxia and reoxygenation following breath-hold dives and overwintering under ice-covered lakes (Jackson, 2002). Exposure to CDH also affects the response to digestion in snapping turtles. Compared with controls, peak postprandial metabolic rates are higher in juvenile turtles exposed to CDH (suggesting an increased metabolic cost of digestion) and this is supported by higher systemic blood flows (Wearing et al., 2017).

The cellular and molecular mechanisms driving cardiovascular programming in reptiles may involve mitochondrial remodelling, as CDH appears to improve mitochondrial efficiency in American alligators and snapping turtles, and this is driven by a lower proton leak (Galli et al., 2016, 2021). Furthermore, CDH induces substantial changes in the cardiac proteome of American alligators prior to hatching, and these changes are largely maintained into juvenile life, with animals from hypoxic incubations showing a shift in protein synthesis (transcription and translation), cellular organization, metabolic adjustments and protein degradation (Alderman et al., 2019). Proteins involved in metabolism are particularly enriched in juvenile alligator hearts from hypoxic incubations, including those with roles in fatty acid oxidation, the citric acid cycle and oxidative phosphorylation (see Glossary). Also worth noting is an increased protein expression of the antioxidant superoxide dismutase, which – in addition to the improved ability to recycle proteins – may help to manage ROS production (Alderman et al., 2019). Finally, we have recently shown that cardiac programming by CDH in snapping turtles is supported by differential expression and DNA methylation of genes associated with sarcomere function, ion-channels, cardiomyocyte survival and heart rate (Ruhr et al., 2021).

In summary, it is clear that CDH programmes the cardiovascular physiology of American alligators and snapping turtles, but in contrast to birds and mammals, the phenotype is not overtly dysfunctional; in fact, in many cases, it appears to be beneficial. The fact that these two species lack many of the pathological signatures associated with CDH (Table S3) suggests the long-term outcome of CDH may be more dependent on body temperature and metabolic rate, rather than phylogeny. It is possible that the higher metabolic costs associated with endothermy place an additional metabolic burden on juvenile and adult birds and mammals exposed to CDH, leading to pathological outcomes.

## Effects of hypoxia on the cardiovascular system of fishes

Among the vertebrate classes, fish are prone to experiencing the most severe levels of hypoxia during development, particularly in climate change scenarios (Box 1). Previous work has shown that CDH alters a wide range of phenotypic traits in teleosts, including metabolic rate (Del Rio et al., 2021), swimming performance (Johnston et al., 2013), sex ratios (Robertson et al., 2014), the balance of sex hormones (Shang and Wu, 2004) and brain development (Mikloska et al., 2022). Nevertheless, surprisingly little is known about the effects of CDH on the teleost cardiovascular system. Comparisons with the other oviparous classes is also difficult because the levels of hypoxia used in fish studies are considerably more severe than those used in studies of reptiles and birds.

### Effects of CDH on growth and cardiac mass in fishes

Similarly to the other vertebrate classes, fish embryos or larvae exposed to CDH have reduced body mass (Table S1A), which renders the individuals less competitive and more vulnerable to predation (Mason, 1969). The growth restriction is driven by the activation of hypoxia inducible factor (HIF), which ultimately supresses the insulin-like growth factor (IGF) pathway (Kajimura et al., 2004; Sun et al., 2011). Fish embryos exposed to CDH also have reduced developmental rates, delayed hatching and delayed heart morphogenesis (Bagatto, 2005; Ciuhandu et al., 2005; Del Rio et al., 2021; Kajimura et al., 2005; Miller et al., 2011, 2008). These effects are particularly prevalent when fish are exposed to hypoxia in the later embryonic stages, which is presumably due to the increasing O_2_ demands of the developing organism and the O_2_-diffusion limitations across the egg membrane (Rombough, 1988).

Although acute hypoxia exposure slows growth and delays development during embryogenesis, upon reoxygenation, hypoxia-exposed embryos often (but not always) return to the same size as control animals (Table S1A). Zebrafish embryos exposed to ∼1–2% O_2_ from 24 to 36 hours post-fertilisation (hpf) are shorter than control animals, but the embryos catch up if they are returned to normoxia (Kamei et al., 2018). The catch-up growth in zebrafish embryos is mediated in part by the IGF pathway (Kamei et al., 2011). Specifically, IGF pathway activity, stimulated by insulin receptor substrate 1 (IRS1)-mediated IGF signalling, helps maintain neural crest cell populations during hypoxia (Kamei et al., 2018). Reductions in neural crest cell numbers – either through ablation or by a combination of hypoxia and reduced IRS1-stimulated IGF signalling – prevents catch-up growth upon reoxygenation in zebrafish (Kamei et al., 2018).

To our knowledge, the effects of CDH on cardiac mass in embryonic/larval fishes have not been directly studied, but there have been measurements of ventricular volume. In zebrafish, hypoxia (3% O_2_) leads to a reduction in ventricular end diastolic and systolic volume at 96 hpf, but an increase at 5 days (Table S1B). This suggests that hypoxia initially causes a reduction in heart size in embryonic zebrafish, but cardiac enlargement occurs once they reach the larval stages. Interestingly, *in vivo* imaging of zebrafish larvae has shown that brain blood flow is unchanged by hypoxia (Schwerte et al., 2003), despite an overall redistribution of blood to the red layer of muscle to enhance O_2_ uptake at 7 days post fertilisation (dpf). This suggests that although blood flow distribution is changed, the brain-sparing effect is absent (El–Fiky and Wieser, 1988). Although these studies have only been performed on one species, it is possible that the brain-sparing effect is unnecessary in fish. Instead, blood is redistributed towards the muscle to enhance O_2_ uptake to the body.

### Effect of CDH on O_2_-carrying capacity and cardiac function in fishes

Like other vertebrates (Galli et al., 2023), embryonic and larval fishes exposed to hypoxia trigger mechanisms to enhance O_2_ extraction. Stage-matched comparisons reveal a greater expression of the higher O_2_ affinity form of embryonic haemoglobin in fish incubated in hypoxia (6% O_2_) compared with those in normoxia (Bianchini and Wright, 2013). Similarly, erythropoiesis is stimulated from 7 dpf in hypoxic zebrafish larvae (Schwerte et al., 2003), and intersegmental blood vessel vascularisation is increased from 6 dpf (Yaqoob and Schwerte, 2010). O_2_ extraction may also be enhanced through the activation of O_2_-sensitive transcription factors, such as HIF. Lake whitefish and zebrafish embryos and larvae show hypoxia-induced, stage-specific changes in the expression of HIF1a and its associated downstream targets, which are known to stimulate haematopoiesis (Wang and Semenza, 1996) and angiogenesis (Iyer et al., 1998), and have been shown to enhance hypoxia tolerance in early life in some studies (Mandic et al., 2020; Robertson et al., 2014; Whitehouse and Manzon, 2019), but not others (Levesque et al., 2019). Finally, behavioural adaptations may also lead to increased O_2_ extraction. For example, hypoxia (3% O_2_) has been shown to induce pectoral fin motions in zebrafish (from 2 dpf) to aid O_2_ uptake (Jonz and Nurse, 2005), and acute hypoxia exposure causes suppression of O_2_ uptake while simultaneously increasing tail beat frequency – potentially in an attempt to reoxygenate the egg case – in little skate embryos (Di Santo et al., 2016).

In addition to increasing O_2_ extraction, embryonic and larval fishes can also increase O_2_ transport to the tissues through alterations in cardiovascular dynamics. During early embryogenesis under normal conditions, fishes rely on diffusion for the supply of O_2_ to their respiring tissues (Burggren, 2004; Grillitsch et al., 2005). This has been demonstrated in developing zebrafish where, prior to ∼14 dpf, reducing the blood's O_2_-carrying capacity elicits no changes in either cardiac output or anaerobic metabolism, implying that under standard conditions, there is no essential role for convective O_2_ (Jacob et al., 2002). However, this is not the case under hypoxic conditions. Zebrafish incubated in hypoxia (∼10% O_2_) display greater heart rate and cardiac output than those in normoxia from 4 dpf onwards, which is likely to increase convective O_2_ transport and act to complement the O_2_ obtained through diffusion to meet the organism's total O_2_ demand (Grillitsch et al., 2005; Jacob et al., 2002). Interestingly, this implies that the afferent nervous system can sense and respond to hypoxia by increasing heart rate from 4 dpf, approximately 10 days before convective O_2_ transport is required under normoxic conditions. These studies suggest that CDH hastens the shift from diffusion to convection-based O_2_ provision in zebrafish embryos (Jacob et al., 2002), but further work is required on this topic. Similarly to embryonic reptiles, there is evidence that these cardiovascular adjustments may improve hypoxia tolerance in the short-term, as *P*_Crit_ is lower in hypoxic zebrafish (Robertson et al., 2014) and Atlantic salmon (Wood et al., 2019b) compared with that of normoxic counterparts.

CDH also causes long-term changes in heart rate in embryonic zebrafish (Table S1D), but the magnitude and direction are variable. In general, tachycardia is the dominant response for embryonic zebrafish exposed to relatively mild or moderate levels of hypoxia (8–10% O_2_) at temperatures of 28–31°C. However, severe hypoxia (2–4% O_2_) causes bradycardia (Table S1D), which is mediated by a release of vagal tone or increase in catecholamines (Steele et al., 2011, 2009). Nevertheless, cardiac output remains constant in chronically hypoxic larval or embryonic zebrafish owing to an elevated stroke volume, and in some cases it is even increased (Cypher et al., 2018; Jacob et al., 2002; Moore et al., 2006; Yaqoob and Schwerte, 2010). Larval zebrafish subjected to hypoxia (4% O_2_) also have significantly increased gene expression of β1, β2a and β2b adrenergic receptors (ARs) at 4 dpf relative to normoxic fish (Steele et al., 2009), and CDH increases cardiac responsiveness to agonists of adrenergic signalling and delays the onset of cholinergic control (see Glossary) in the rainbow trout (Miller et al., 2011). However, sympathetic sensitisation in zebrafish is likely to be dependent on the duration of hypoxia exposure and developmental stage, as the expression of β1AR does not change in whole zebrafish embryos (2 dpf) exposed to only 12 h or 24 h of hypoxia (5% O_2_; Ton et al., 2002, 2003).

### The long-term effects of CDH on fish growth and the cardiovascular system

Despite the ecological importance, the long-term effects of CDH are poorly studied in fishes, and the results are highly variable. Trout larvae exposed to CDH exhibit catch-up growth with a significantly greater increase in weight (278% for CDH versus 188% for control) and length (64% for CDH versus 27% for control), eventually leading to significantly larger fry body weights and lengths compared with controls (Johnston et al., 2013). In contrast, juvenile Chinook salmon and European seabass exposed to CDH during embryogenesis are significantly smaller than controls (Del Rio et al., 2019), and growth restriction in hypoxic zebrafish embryos also persists into adulthood (Table S1A). However, no effect of CDH has been found on body weight in adult Atlantic salmon (Wood et al., 2017). Collectively, these studies show that the long-term effect of CDH on body mass is extremely variable in teleosts, and it depends on multiple factors, including species and body temperature.

To our knowledge, nothing is known about the long-term effects of CDH on juvenile and adult teleost cardiac structure or function. However, there is evidence of differential cardiac gene expression in rainbow trout exposed to CDH, including that of the common housekeeping genes 18S ribosomal RNA and acidic ribosomal phosphoprotein, and protein expression of cardiac troponin I (Johnston et al., 2013). Furthermore, previous work has shown that zebrafish cardiac morphology can be altered by other environmental stressors during development, including temperature and CO_2_ (see below), as well as crude oil and polycyclic aromatic hydrocarbons (for a review, see Takeshita et al., 2021). Therefore, there is ample evidence that the fish heart is capable of developmental plasticity, but there is a distinct lack of studies on CDH.

Although few studies have explicitly investigated hypoxic programming in the fish heart, several studies address aspects of whole-organism performance and fitness that potentially link to cardiac performance. Hypoxic-incubated (10% O_2_) rainbow trout show a consistently lower maximum relative swimming speed than normoxic controls across three developmental stages, which is thought to be caused by a delay in cardiac maturation (Johnston et al., 2013). Zebrafish and Atlantic salmon larvae exposed to CDH also show an improved whole-animal hypoxia tolerance. However, this phenotype does not persist into the juvenile and adult life stages (Del Rio et al., 2021; Robertson et al., 2014; Vanderplancke et al., 2015; Wood et al., 2019a; Wood et al., 2017). In fact, European seabass larvae raised in hypoxia (8% O_2_) show a reduced hypoxia tolerance as juveniles, which is associated with an increased prevalence of opercular abnormalities (Cadiz et al., 2017). Similarly, 15-month-old Atlantic salmon exposed to CDH (10% O_2_) are marginally less hypoxia tolerant than normoxia-incubated animals, although their aerobic scope is similar (Wood et al., 2017), and there is no effect of CDH (10% O_2_) on hypoxia tolerance in juvenile Chinook salmon (Del Rio et al., 2021). Nevertheless, the physiological response to hypoxia can be affected by CDH in some fish. For example, when seabass are exposed to hypoxia as juveniles, fish that experienced hypoxia during embryogenesis show changes in haemoglobin subtype expression, but no differences in overall haemoglobin concentration (Cadiz et al., 2017).

## Climate change and the interactive effects of CDH with other stressors

Oviparous vertebrates rarely experience CDH in isolation because other developmental stressors often occur simultaneously (Box 2). Indeed, under natural conditions, CDH often occurs alongside fluctuations in CO_2_, temperature, pH and salinity. Given that climate change is increasing the magnitude and frequency of these events, it is becoming increasingly important to study these interactive effects.

### Interactive effects of CDH and temperature

Although maternal nest choice and behaviour may partly shield terrestrial embryos from thermal stress, recent models suggest that global warming will increase the incubation temperatures of avian and reptilian eggs (Du et al., 2023; DuRant et al., 2019). Extensive research has shown that thermal stress can dramatically alter the morphology and physiology of reptilian and avian embryos, including changes in growth, body mass, cardiac mass, heart rate, mitochondrial density and respiration (Ben-Ezra and Burness, 2017; Du et al., 2023; Du and Shine, 2015; Du et al., 2010; Singh et al., 2020). Although data are scarce, some studies have investigated the interactive effects of temperature and hypoxia in avian and reptilian embryos. For example, Lourens et al. (2007) undertook a study in chickens where incubation temperature was increased from 37.8°C to 38.9°C at either 17% or 21% O_2_. Temperature and hypoxia had independent effects on hatch time, body weight, yolk-free body weight and relative heart weight; however, there were no interactions between O_2_ and temperature (Lourens et al., 2007). Another study in chickens found that mild levels of hypoxia (17% O_2_) did not produce any effects on embryonic body mass or heart mass, even when temperature was increased from 37.8 or 38.9°C (Table S1A). By contrast, the negative effects of hypobaric hypoxia (2877 m, 15% O_2_ SLE) during embryonic development on body mass, swimming speed and heart rate in adult viperine snakes at 28°C disappear when temperature is reduced to 24°C (Souchet et al., 2020a,b). Interestingly, increasing the temperature to 32°C produces a completely different phenotype, with a reduced heart rate, smaller body mass and faster swimming speed. The surprising improvement in swimming performance in adult snakes at high altitude at the warmest temperature persisted after relocation to low elevation (Souchet et al., 2020a). The authors suggest that constraints on development may be offset by the preservation of performance traits (perhaps through cardiorespiratory plasticity). Collectively, these studies suggest that the vertical colonisation potential of reptiles and birds (see below) will be affected by the interaction between temperature and O_2_ availability.

Interestingly, a recent meta-analysis found that aquatic embryonic ectotherms are more than three times as plastic as terrestrial ectotherms when exposed to thermal stress during development (Pottier et al., 2022). Indeed, a large body of literature has shown that an increase in developmental temperature affects embryonic and larval fish growth rate, sex ratio, body size, metabolism, heart rate, cardiac morphology, hypoxia tolerance and swimming performance (Dimitriadi et al., 2018; Eme et al., 2015; Melendez and Mueller, 2021; Mueller et al., 2011; Pelster, 1999; Vagner et al., 2019; Zambonino-Infante et al., 2013). Some of these studies found effects that lasted into adulthood, including increased ventricular roundness in juvenile and adult male zebrafish exposed to elevated temperatures during embryogenesis (Dimitriadi et al., 2018, 2021). However, the short- and long-term effects of developmental temperature are highly variable in fish, and interestingly, the same meta-analysis found that persistent effects on thermal tolerance limits in adulthood were surprisingly weak (Pottier et al., 2022). Whether the same is true when elevated temperature occurs in combination with hypoxia is largely unknown, because surprisingly little is known about this interaction. One study on Chinook salmon investigated developmental outcomes in fish that were reared from fertilization to the fry stage at two temperatures (10°C and 14°C) and two O_2_ levels (100% or 50% air saturation). Although temperature and O_2_ saturation had independent effects on growth and acute hypoxia tolerance, there was no interaction between the two stressors (Del Rio et al., 2019). This was also the case in European sea bass exposed to different temperature and hypoxia combinations (40% or 100% air saturation×15°C and 20°C) from the flexion stage until the end of larval development (Cadiz et al., 2018). However, there were significant interactions on hatching success and thermal tolerance in Chinook salmon, with higher temperature generally potentiating the effects of hypoxia (Del Rio et al., 2019). Lastly, CDH causes an increase in cardiac output and heart rate in zebrafish embryos at 25–31°C, but the magnitude of the response is lowest at 31°C, presumably because the fish had neared their maximal cardiovascular capacity (Jacob et al., 2002). Clearly, more studies are warranted and necessary to understand the physiological implications of temperature and hypoxia interactions during development.

### Climate-driven elevational range shifts and high-altitude acclimatization

Global warming is driving some reptilian and avian species to shift their geographical distributions towards higher-elevation habitats with lower O_2_ availability (Neate-Clegg and Tingley, 2023; Rubenstein et al., 2023). Developmental plasticity will therefore play a pivotal role in successful colonization of high-altitude environments. One approach to predicting the effects of climate-driven elevational range shifts is the so-called ‘transplant’ experiment, whereby gravid females or embryos from one elevation are transported and maintained at another. In this regard, recent work on the viperine snake has been particularly insightful, because this species has repeatedly migrated across elevational gradients to colonise high-altitude environments, in association with historical warming and cooling cycles (Gómez and Lunt, 2007). Transplanting viperine snake embryos at 28°C from 436 m (20% O_2_ SLE) to 2877 m (15% O_2_ SLE) increases heart rate, reduces body mass and decreases swimming ability (Souchet et al., 2020b). Importantly, post-hatching reciprocal transplant of snakes back to 436 m does not fully recover swimming performance, and the response is significantly temperature sensitive (see ‘Interactive effects of CDH and temperature’, above). Similar results were found in common wall lizards, where transplantation of embryos from sea level to 2877 m (15–16% O_2_ SLE) leads to suppressed embryonic metabolism, cardiac hypertrophy and larger eggs that produce hatchlings with relatively low mass (Cordero et al., 2017). In contrast, transplantation of lowland Mongolia racerunner lizards to 2036 m (16–17 O_2_ SLE) had no effect on embryonic development (hatching time and success) or hatchling phenotypes (body size and locomotor performance), which suggests this species can buffer the impact of hypobaric hypoxia (Li et al., 2020).

Another approach to predicting the effects of climate-driven elevational range shifts is to compare embryonic outcomes in native highland versus native lowland individuals from the same species. These types of studies reveal genetic adaptations that arise over successive generations. Perhaps unsurprisingly, numerous studies have clearly shown that embryonic highland oviparous species are less sensitive to hypoxia than their lowland counterparts. For example, there is no effect of 12% O_2_ exposure on embryonic body weight in geese raised at high altitude (1600 m, Table S1A), and hatchling masses of high-altitude coots (4100 m) are similar or slightly greater than those at sea-level (Carey et al., 1989). Native high-altitude ptarmigan and coot embryos (4200 m; León-Velarde and Monge-C, 2004) and bar-headed goose embryos (Snyder et al., 1984) have a greater O_2_-carrying capacity than their sea-level counterparts, with increased haematocrit, haemoglobin, capillary density and blood O_2_ affinity. Adult fishes from high-altitude habitats in China also possess adaptations related to haemoglobin, as well as expansions of gene families associated with energy metabolism, ion transport and the response to hypoxia (Kang et al., 2017; Lei et al., 2021; Tong et al., 2017). Lastly, cardiac citrate synthase activity in white-tailed ptarmigan (4200 m) is higher than that of its sea level counterparts, suggesting increased mitochondrial density and oxidative capacity (Carey and Martin, 1997). These studies demonstrate that prolonged high-altitude residence in oviparous vertebrates confers some protection against hypobaric hypoxia (similarly to humans; Giussani et al., 2001), and this is associated with adaptations in both O_2_-carrying capacity and utilisation. Nevertheless, living at high altitude for six successive generations does not completely protect chicken embryos from the effects of hypobaric hypoxia. Growth restriction in chickens is improved by high-altitude residence, but there is still a significant reduction in embryonic body mass with hypobaric hypoxia, as well as cardiac hypertrophy, ventricular wall thickening, aortic medial thickening and an increase in adrenal catecholamines (Giussani et al., 2007; Salinas et al., 2010, 2011). The effects can be prevented if high-altitude hens are given O_2_ supplementation, which confirms that hypoxia rather than hypobaria is driving the cardiovascular response. Furthermore, the effects persist into adulthood when chickens are maintained at high altitude for a further 6 months, and there is also evidence of pulmonary hypertension, right-sided heart dysfunction and hypotension (Herrera et al., 2013; Salinas et al., 2014). Interestingly, American alligators exposed to CDH and maintained in hypoxia into juvenile life also have signs of pulmonary hypertension, including a decreased ratio of the right ventricle to left ventricle (Owerkowicz et al., 2009). Collectively, these studies suggest some of the problems associated with CDH in chickens cannot be prevented by residence at high altitude (at least across six generations) and post-hatch exposure to hypoxia may cause further damage, including pulmonary hypertension. Whether later generations would eventually evolve better protection awaits investigation.

### Potential interactive effects of CDH and CO_2_ concentration

Despite the fact that oviparous vertebrates often experience hypoxia and hypercapnia simultaneously (both naturally and in climate change scenarios; Box 2), we are unaware of any studies that have investigated the combined effects of CDH and chronic hypercapnia. There are however, several studies that have shown interactive effects of acute hypoxia and hypercapnia (<1 day) on chick embryonic O_2_-carrying capacity and acid–base balance (Andrewartha et al., 2011, 2014; Burggren et al., 2023, 2012; Mueller et al., 2017). Furthermore, there is ample evidence that embryonic growth and cardiovascular outcomes can be affected by chronic hypercapnia alone, even at physiological levels. For example, exposure of embryonic chickens and ducks to CO_2_ concentrations that they would normally encounter in the nest (1%) or higher (4%) increases body mass, compared with atmospheric levels (0.004%) and this effect persists into adulthood (De Smit et al., 2006; El-Hanoun et al., 2019; Everaert et al., 2007; Fares et al., 2012; Verhoelst et al., 2011). A similar observation has been made in common snapping turtles exposed to 3.5% CO_2_ (Wearing et al., 2014), and American alligator embryos exposed to 3.5% and 7% CO_2_ have increased relative heart mass and reduced arterial blood pressure (Eme and Crossley, 2015). Lastly, embryonic chickens and ducks exposed to 1% CO_2_ have increased embryonic haemoglobin, packed cell volume (i.e the proportion of blood made up of cells) and red blood cell count (El-Hanoun et al., 2019; Fares et al., 2012). Collectively, these studies suggest that hypercapnia during development could offset hypoxic growth restriction in birds and reptiles, and it could potentiate some of the cardiovascular responses to hypoxia.

A large body of evidence suggests that juvenile and adult fish possess sufficient acid–base and osmoregulatory capabilities to tolerate very high CO_2_ levels (>2000 µatm; Murray et al., 2016). However, a recent metanalysis confirmed that fish embryos and larvae are significantly more sensitive to hypercapnia than their adult counterparts (Cattano et al., 2018). Indeed, embryonic or larval fish have significantly higher levels of mortality and reduced growth at *P*_CO_2__ levels consistent with climate change projections (∼1000 atm). The increased sensitivity is likely due to ontogenic differences in respiration modes (dermal versus gills) and insufficient acid–base regulation prior to gill formation (Ishimatsu et al., 2008). There is also evidence that chronic hypercapnia affects cardiac function in some larval fish species. Chronic exposure to *P*_CO_2__ at ∼1100–1300 μatm causes tachycardia in Pacific herring, garfish and zebrafish larvae (Alter and Peck, 2021; Miller, 2013; Villalobos et al., 2020). However, numerous other studies have found no effect of hypercapnia on growth, heart rate, haemoglobin and mitochondrial function, and some have even found increased growth (Esbaugh, 2018; Leo et al., 2018; Mu et al., 2015; Scheuffele, 2017; Sun et al., 2019). Therefore, although there is certainly a case to study the interaction between hypercapnia and hypoxia in fish embryos and larvae, the effects may be relatively modest compared with those of temperature.

## Conclusions and perspectives

Oviparous ectotherms produce viable young when eggs are exposed to CDH, but there are numerous effects on the cardiovascular system at multiple levels of biological organisation, both during development and in postnatal life (Fig. 1). Despite vastly different cardiac designs and body temperatures, the embryonic cardiovascular responses are generally well conserved among vertebrates, and include asymmetric growth restriction, relative cardiac enlargement, alterations in heart rate, enhanced sympathetic activity and an increase in O_2_-carrying capacity. In the long term, these phenotypic changes programme cardiovascular abnormalities in chickens that are very similar to those of mammals, leading to reduced cardiac performance and pathological cardiovascular signatures. The impact of CDH in American alligators and snapping turtles is less severe in juvenile life and may even be beneficial under circumstances of increased physiological stress. This suggests that the increased metabolic demand associated with endothermy places an additional burden on the avian and mammalian heart.

Unsurprisingly, the embryonic and postnatal response to CDH depends on the severity of hypoxia. In birds and reptiles, most responses are only evident at O_2_ concentrations at or below 15% saturation. These levels of O_2_ are commonly experienced by many embryonic reptilian species, which suggests that CDH is a significant driver of individual variation. In contrast, most lowland embryonic avians are unlikely to experience O_2_ concentrations below 20% O_2_, which makes CDH less ecologically relevant. However, megapode species develop at O_2_ concentrations below 15%, so it would be interesting to see whether these species are uniquely adapted to hypoxia. The situation in fishes is far more complex, and there does not seem to be any obvious O_2_ threshold for a cardiovascular response, even within the same species. This is probably because the levels of CDH are much more severe in the fish studies (45–95% reduction in O_2_) versus the avian and reptilian studies (20–50% reduction in O_2_), probably leading to higher levels of variation, and making comparisons between these groups complicated.

More work needs to be done to characterise the phenotypic responses and thresholds for CDH in the presence of other stressors, such as hypercapnia and temperature in all oviparous vertebrate groups. Interestingly, hypercapnia alone appears to have both synergistic and antagonistic responses to hypoxia in oviparous vertebrates, which means that the combination of these two stressors is expected to produce entirely different phenotypes. This is relevant to normal development because reptiles and birds experience hypoxia and hypercapnia simultaneously, and most studies use non-physiological levels of CO_2_ when investigating hypoxia. It is also important in the context of climate change because the prevalence and intensity of hypercapnia is increasing, particularly in aquatic environments. Unsurprisingly, warming temperatures exacerbate the effects of developmental hypoxia in some oviparous species, which is concerning considering global warming and the increased prevalence and intensity of heat waves. The timing of extreme weather events is also crucial, because most species possess critical windows in development where the cardiovascular system is especially sensitive to stress. Furthermore, we expect species with faster developmental rates and shorter gestations to be disproportionately affected by heat waves and extreme weather events, compared with longer-gestation species, because a greater proportion of their development will be affected. Obviously, the challenge is to study the integrative effects of CDH, hypercapnia and warming on embryonic and adult phenotypic outcomes. In this regard, it is also critically important to gather accurate data about the effects of climate change on nest gas tensions and temperatures.

Future work should also focus on transplantation studies to determine the effects of high-altitude acclimation on reptilian and avian developmental outcomes. Studies like these are important because the phenotypic response to high-altitude hypoxia in lowland species will ultimately determine the colonization potential of these animals as the planet continues to warm. From the limited data available, it is clear that reptiles and birds respond to hypobaric hypoxia in a similar fashion to isobaric hypoxia, and some of the traits cannot be reversed by returning the animals to sea level. Long-term residence at high altitude affords protection in most avian and reptilian species, but domestic chickens raised at high altitude for six generations still undergo some level of growth restriction and cardiac remodelling in response to CDH. Importantly, the phenotype worsens with continued exposure to hypoxia post-hatch. Clearly, more multigenerational studies are necessary to understand the impact of cardiovascular plasticity on the vertical colonisation potential of oviparous birds and reptiles.

Lastly, there are some questions in this field that are almost completely unstudied. For example, our understanding of the effects of CDH and other stressors on the amphibian cardiovascular system is severely lacking. This is surprising, as this class of vertebrates is one of the most likely to experience fluctuations in developmental O_2_, CO_2_ and temperature (Box 2). There is also very little known about sex-dependent differences in the response to CDH among oviparous vertebrates. It is well established in the mammalian literature that cardiometabolic responses to developmental stressors are strongly sex-dependent, with females often being protected against detrimental long-term health outcomes compared to males (Giussani, 2021; Sandovici et al., 2022). Sex-dependent differences have been observed in some avian studies, but these effects are largely unstudied in ectothermic vertebrates. Similarly, the transgenerational effects of CDH and the underlying epigenetic mechanisms are very poorly studied in oviparous vertebrates. In this regard, several studies have shown that parental exposure to hypoxia can improve hypoxia tolerance in zebrafish offspring (Burggren, 2014; Ragsdale et al., 2022). These kinds of phenomena are particularly important to study, because transgenerational plasticity will play a crucial role in determining a species' ability to cope with a rapidly changing environment (Donelson et al., 2018).

## Supplementary Material

10.1242/jexbio.245530_sup1 Supplementary information

## References

[JEB245530C1] Akiyama, R., Mitsubayashi, H., Tazawa, H. and Burggren, W. W. (1999). Heart rate responses to altered ambient oxygen in early (days 3-9) chick embryos in the intact egg. *J. Comp. Physiol. B Biochem. Syst. Eenviron. Physiol.* 169, 85-92. 10.1007/s003600050197 10227182

[JEB245530C2] Alderman, S. L., Crossley, D. A., Elsey, R. M. and Gillis, T. E. (2019). Hypoxia-induced reprogramming of the cardiac phenotype in American alligators (*Alligator mississippiensis*) revealed by quantitative proteomics. *Sci. Rep.* 9, 8592. 10.1038/s41598-019-45023-3 31197188 PMC6565670

[JEB245530C3] Alter, K. and Peck, M. A. (2021). Ocean acidification but not elevated spring warming threatens a European seas predator. *Sci. Total Environ.* 782, 146926. 10.1016/j.scitotenv.2021.146926

[JEB245530C4] Altimiras, J. and Phu, L. (2000). Lack of physiological plasticity in the early chicken embryo exposed to acute hypoxia. *J. Exp. Zool.* 286, 450-456. 10.1002/(SICI)1097-010X(20000401)286:5<450::AID-JEZ2>3.0.CO;2-Y 10684568

[JEB245530C5] Andrewartha, S. J., Tazawa, H. and Burggren, W. W. (2011). Embryonic control of heart rate: examining developmental patterns and temperature and oxygenation influences using embryonic avian models. *Respir. Physiol. Neurobiol.* 178, 84-96. 10.1016/j.resp.2011.04.014 21530689

[JEB245530C6] Andrewartha, S. J., Tazawa, H. and Burggren, W. W. (2014). Acute regulation of hematocrit and acid-base balance in chicken embryos in response to severe intrinsic hypercapnic hypoxia. *Respir. Physiol. Neurobiol.* 195, 1-10. 10.1016/j.resp.2014.01.019 24509299

[JEB245530C7] Asson-Batres, M. A., Stock, M. K., Hare, J. F. and Metcalfe, J. (1989). O_2_ effect on composition of chick embryonic heart and brain. *Respir. Physiol.* 77, 101-109. 10.1016/0034-5687(89)90033-9 2552550

[JEB245530C8] Bagatto, B. (2005). Ontogeny of cardiovascular control in zebrafish (*Danio rerio*): effects of developmental environment. *Comp. Biochem. Physiol. A Mol. Integr. Physiol.* 141, 391-400. 10.1016/j.cbpb.2005.07.002 16085439

[JEB245530C9] Bautista, N. M., Petersen, E. E., Jensen, R. J., Natarajan, C., Storz, J. F., Crossley, D. A. 2nd and Fago, A. (2021). Changes in hemoglobin function and isoform expression during embryonic development in the American alligator, *Alligator mississippiensis*. *Am. J. Physiol. Regul. Integr. Comp. Physiol.* 321, R869-R878. 10.1152/ajpregu.00047.2021 34704846 PMC8714809

[JEB245530C10] Ben-Ezra, N. and Burness, G. (2017). Constant and cycling incubation temperatures have long-term effects on the morphology and metabolic rate of Japanese quail. *Physiol. Biochem. Zool.* 90, 96-105. 10.1086/688383 28051937

[JEB245530C11] Benthuysen, J. A., Oliver, E. C., Chen, K. and Wernberg, T. (2020). Advances in understanding marine heatwaves and their impacts. *Front. Mar. Sci.* 7, 147. 10.3389/fmars.2020.00147

[JEB245530C12] Bianchini, K. and Wright, P. A. (2013). Hypoxia delays hematopoiesis: retention of embryonic hemoglobin and erythrocytes in larval rainbow trout, Oncorhynchus mykiss, during chronic hypoxia exposure. *J. Exp. Biol.* 216, 4415-4425. 10.1242/jeb.083337 24031065

[JEB245530C13] Brusatte, S. L., Benton, M. J., Desojo, J. B. and Langer, M. C. (2010). The higher-level phylogeny of Archosauria (Tetrapoda: Diapsida). *J. Syst. Palaeontol.* 8, 3-47. 10.1080/14772010903537732

[JEB245530C14] Burggren, W. W. (2004). What is the purpose of the embryonic heart beat? Or how facts can ultimately prevail over physiological dogma. *Physiol. Biochem. Zool.* 77, 333-345. 10.1086/422230 15295688

[JEB245530C15] Burggren, W. W. (2014). Epigenetics as a source of variation in comparative animal physiology – or – Lamarck is lookin’ pretty good these days. *J. Exp. Biol.* 217, 682-689. 10.1242/jeb.086132 24574384

[JEB245530C16] Burggren, W. W., Andrewartha, S. J. and Tazawa, H. (2012). Interactions of acid–base balance and hematocrit regulation during environmental respiratory gas challenges in developing chicken embryos (*Gallus gallus*). *Respir. Physiol. Neurobiol.* 183, 135-148. 10.1016/j.resp.2012.06.011 22709561

[JEB245530C17] Burggren, W., Filogonio, R. and Wang, T. (2020). Cardiovascular shunting in vertebrates: a practical integration of competing hypotheses. *Biol. Rev. Camb. Philos. Soc.* 95, 449-471. 10.1111/brv.12572 31859458

[JEB245530C18] Burggren, W. W., Andrewartha, S. J., Mueller, C. A., Dubansky, B. and Tazawa, H. (2023). Acid-base and hematological regulation in chicken embryos during internal progressive hypercapnic hypoxia. *Respir. Physiol. Neurobiol.* 308, 103996. 10.1016/j.resp.2022.103996 36402363

[JEB245530C19] Cadiz, L., Servili, A., Quazuguel, P., Madec, L., Zambonino-Infante, J. L. and Mazurais, D. (2017). Early exposure to chronic hypoxia induces short- and long-term regulation of hemoglobin gene expression in European sea bass (*Dicentrarchus labrax*). *J. Exp. Biol.* 220, 3119-3126. 10.1242/jeb.160713 28646037

[JEB245530C20] Cadiz, L., Ernande, B., Quazuguel, P., Servili, A., Zambonino-Infante, J.-L. and Mazurais, D. (2018). Moderate hypoxia but not warming conditions at larval stage induces adverse carry-over effects on hypoxia tolerance of European sea bass (*Dicentrarchus labrax*) juveniles. *Mar. Environ. Res.* 138, 28-35. 10.1016/j.marenvres.2018.03.011 29628391

[JEB245530C21] Cai, W.-J., Hu, X., Huang, W.-J., Murrell, M. C., Lehrter, J. C., Lohrenz, S. E., Chou, W.-C., Zhai, W., Hollibaugh, J. T., Wang, Y. et al. (2011). Acidification of subsurface coastal waters enhanced by eutrophication. *Nat. Geosci.* 4, 766-770. 10.1038/ngeo1297

[JEB245530C22] Carey, C. and Martin, K. (1997). Physiological ecology of incubation of ptarmigan eggs at high and low altitudes. *Wildl. Biol.* 3, 211-218. 10.2981/wlb.1997.026

[JEB245530C23] Carey, C., Leon-Velarde, F., Dunin-Borkowski, O., Bucher, T. L., de la Torre, G., Espinoza, D. and Monge, C. (1989). Variation in eggshell characteristics and gas exchange of montane and lowland coot eggs. *J. Comp. Physiol. B* 159, 389-400. 10.1007/BF00692411

[JEB245530C24] Cattano, C., Claudet, J., Domenici, P. and Milazzo, M. (2018). Living in a high CO_2_ world: a global meta–analysis shows multiple trait–mediated fish responses to ocean acidification. *Ecol. Monogr.* 88, 320-335. 10.1002/ecm.1297

[JEB245530C25] Ciuhandu, C. S., Stevens, E. D. and Wright, P. A. (2005). The effect of oxygen on the growth of *Oncorhynchus mykiss* embryos with and without a chorion. *J. Fish Biol.* 67, 1544-1551. 10.1111/j.1095-8649.2005.00856.x

[JEB245530C26] Cordero, G. A., Andersson, B. A., Souchet, J., Micheli, G., Noble, D. W. A., Gangloff, E. J., Uller, T. and Aubret, F. (2017). Physiological plasticity in lizard embryos exposed to high-altitude hypoxia. *J. Exp. Zool. Part A: Ecol. Integr. Physiol.* 327, 423-432. 10.1002/jez.2115 29356444

[JEB245530C27] Corona, T. B. and Warburton, S. J. (2000). Regional hypoxia elicits regional changes in chorioallantoic membrane vascular density in alligator but not chicken embryos. *Comp. Biochem. Physiol. A Mol. Integr. Physiol.* 125, 57-61. 10.1016/S1095-6433(99)00159-2 10779731

[JEB245530C28] Crossley, D. A. and Altimiras, J. (2005). Cardiovascular development in embryos of the American alligator *Alligator mississippiensis*: effects of chronic and acute hypoxia. *J. Exp. Biol.* 208, 31-39. 10.1242/jeb.01355 15601875

[JEB245530C29] Crossley, D. A., Burggren, W. W. and Altimiras, J. (2003). Cardiovascular regulation during hypoxia in embryos of the domestic chicken *Gallus gallus*. *Am. J. Physiol. Regul. Integr. Comp. Physiol.* 284, R219-R226. 10.1152/ajpregu.00654.2001 12388452

[JEB245530C30] Crossley, D. A. 2nd, Ling, R., Nelson, D., Gillium, T., Conner, J., Hapgood, J., Elsey, R. M. and Eme, J. (2017). Metabolic responses to chronic hypoxic incubation in embryonic American alligators (*Alligator mississippiensis*). *Comp. Biochem. Physiol. A Mol. Integr. Physiol.* 203, 77-82. 10.1016/j.cbpa.2016.08.017 27584614

[JEB245530C31] Crossley, J. L., Lawrence, T., Tull, M., Elsey, R. M., Wang, T. and Crossley, A. C. 2nd. (2022). Developmental oxygen preadapts ventricular function of juvenile American alligators, *Alligator mississippiensis*. *Am. J. Physiol. Regul. Integr. Comp. Physiol.* 323, R739-R748. 10.1152/ajpregu.00059.2022 36121144

[JEB245530C32] Crossley, J. L., Smith, B., Tull, M., Elsey, R. M., Wang, T. and Crossley, D. A. 2nd. (2023). Hypoxic incubation at 50% of atmospheric levels shifts the cardiovascular response to acute hypoxia in American alligators, *Alligator mississippiensis*. *J. Comp. Physiol. B* 193, 545-556. 10.1007/s00360-023-01510-8 37615772

[JEB245530C33] Cypher, A. D., Fetterman, B. and Bagatto, B. (2018). Vascular parameters continue to decrease post-exposure with simultaneous, but not individual exposure to BPA and hypoxia in zebrafish larvae. *Comp. Biochem. Physiol. C Toxicol. Pharmacol.* 206-207, 11-16. 10.1016/j.cbpc.2018.02.002 29454160

[JEB245530C34] De Smit, L., Bruggeman, V., Tona, J. K., Debonne, M., Onagbesan, O., Arckens, L., De Baerdemaeker, J. and Decuypere, E. (2006). Embryonic developmental plasticity of the chick: increased CO_2_ during early stages of incubation changes the developmental trajectories during prenatal and postnatal growth. *Comp. Biochem. Physiol. Part A: Mol. Integr. Physiol.* 145, 166-175. 10.1016/j.cbpa.2006.06.046 16928458

[JEB245530C35] Del Rio, A. M., Davis, B. E., Fangue, N. A. and Todgham, A. E. (2019). Combined effects of warming and hypoxia on early life stage Chinook salmon physiology and development. *Conserv. Physiol.* 7, coy078. 10.1093/conphys/coy078 30834124 PMC6387995

[JEB245530C36] Del Rio, A. M., Mukai, G. N., Martin, B. T., Johnson, R. C., Fangue, N. A., Israel, J. A. and Todgham, A. E. (2021). Differential sensitivity to warming and hypoxia during development and long-term effects of developmental exposure in early life stage Chinook salmon. *Conserv. Physiol.* 9, coab054. 10.1093/conphys/coab054 34257996 PMC8271147

[JEB245530C176] Dhiyebi, H., O'Donnell, M. and Wright, P. (2013). Water chemistry in the microenvironment of rainbow trout Oncorhynchus mykiss embryos is affected by development, the egg capsule and crowding. *J. Fish Biol.* 82, 444-457. 23398061 10.1111/j.1095-8649.2012.03491.x

[JEB245530C37] Di Santo, V., Tran, A. H. and Svendsen, J. C. (2016). Progressive hypoxia decouples activity and aerobic performance of skate embryos. *Conserv. Physiol.* 4, cov067. 10.1093/conphys/cov067 27293746 PMC4732404

[JEB245530C38] Dimitriadi, A., Beis, D., Arvanitidis, C., Adriaens, D. and Koumoundouros, G. (2018). Developmental temperature has persistent, sexually dimorphic effects on zebrafish cardiac anatomy. *Sci. Rep.* 8, 8125. 10.1038/s41598-018-25991-8 29802254 PMC5970236

[JEB245530C39] Dimitriadi, A., Geladakis, G. and Koumoundouros, G. (2021). 3D heart morphological changes in response to developmental temperature in zebrafish: more than ventricle roundness. *J. Morphol.* 282, 80-87. 10.1002/jmor.21283 33617037

[JEB245530C40] Donelson, J. M., Salinas, S., Munday, P. L. and Shama, L. N. S. (2018). Transgenerational plasticity and climate change experiments: where do we go from here? *Glob. Change Biol.* 24, 13-34. 10.1111/gcb.13903 29024256

[JEB245530C41] Doody, J. S. and Refsnider, J. M. (2022). Nesting in reptiles: natural and anthropogenic threats and evolutionary responses. *Front. Ecol. Evol.* 10, 1103193. 10.3389/fevo.2022.1103193

[JEB245530C42] Du, W.-G. and Shine, R. (2015). The behavioural and physiological strategies of bird and reptile embryos in response to unpredictable variation in nest temperature. *Biol. Rev.* 90, 19-30. 10.1111/brv.12089 24593133

[JEB245530C43] Du, W. G., Ye, H., Zhao, B., Warner, D. A. and Shine, R. (2010). Thermal acclimation of heart rates in reptilian embryos. *PLoS One* 5, e15308. 10.1371/journal.pone.0015308 21179473 PMC3001871

[JEB245530C44] Du, W. G., Shine, R., Ma, L. and Sun, B. J. (2019). Adaptive responses of the embryos of birds and reptiles to spatial and temporal variations in nest temperatures. *Proc. Biol. Sci.* 286, 20192078. 10.1098/rspb.2019.2078 31744441 PMC6892042

[JEB245530C45] Du, W.-G., Li, S.-R., Sun, B.-J. and Shine, R. (2023). Can nesting behaviour allow reptiles to adapt to climate change? *Philos. Trans. R. Soc. B: Biol. Sci.* 378, 20220153. 10.1098/rstb.2022.0153 PMC10331901 37427463

[JEB245530C46] DuRant, S. E., Willson, J. D. and Carroll, R. B. (2019). Parental effects and climate change: will avian incubation behavior shield embryos from increasing environmental temperatures? *Integr. Comp. Biol.* 59, 1068-1080. 10.1093/icb/icz083 31168619

[JEB245530C47] Dzialowski, E. M., von Plettenberg, D., Elmonoufy, N. A. and Burggren, W. W. (2002). Chronic hypoxia alters the physiological and morphological trajectories of developing chicken embryos. *Comp. Biochem. Physiol. Part A: Mol. Integr. Physiol.* 131, 713-724. 10.1016/S1095-6433(02)00009-0 11897182

[JEB245530C48] Earhart, M. L., Blanchard, T. S., Harman, A. A. and Schulte, P. M. (2022). Hypoxia and high temperature as interacting stressors: will plasticity promote resilience of fishes in a changing world? *Biol. Bull.* 243, 149-170. 10.1086/722115 36548973

[JEB245530C49] El–Fiky, N. and Wieser, W. (1988). Life styles and patterns of development of gills and muscles in larval cyprinids (Cyprinidae; Teleostei). *J. Fish Biol.* 33, 135-145. 10.1111/j.1095-8649.1988.tb05455.x

[JEB245530C50] El-Hanoun, A., El-Sabrout, K., Abdella, M. and Eid, M. (2019). Effect of carbon dioxide during the early stage of duck egg incubation on hatching characteristics and duckling performance. *Physiol. Behav.* 208, 112582. 10.1016/j.physbeh.2019.112582 31220515

[JEB245530C51] Eme, J. and Crossley, D. (2015). Chronic hypercapnic incubation increases relative organ growth and reduces blood pressure of embryonic American alligator (*Alligator mississippiensis*). *Comp. Biochem. Physiol. Part A: Mol. Integr. Physiol.* 182, 53-57. 10.1016/j.cbpa.2014.12.009 25499241

[JEB245530C52] Eme, J., Crossley, D. A. 2nd and Hicks, J. W. (2011a). Role of the left aortic arch and blood flows in embryonic American alligator (*Alligator mississippiensis*). *J. Comp. Physiol. B Biochem. Syst. Eenviron. Physiol.* 181, 391-401. 10.1007/s00360-010-0494-6 PMC3058339 21053004

[JEB245530C53] Eme, J., Hicks, J. W. and Crossley, D. A. 2nd. (2011b). Chronic hypoxic incubation blunts a cardiovascular reflex loop in embryonic American alligator (*Alligator mississippiensis*). *J. Comp. Physiol. B Biochem. Syst. Eenviron. Physiol.* 181, 981-990. 10.1007/s00360-011-0569-z 21445563

[JEB245530C54] Eme, J., Rhen, T., Tate, K. B., Gruchalla, K., Kohl, Z. F., Slay, C. E. and Crossley, D. A. 2nd. (2013). Plasticity of cardiovascular function in snapping turtle embryos (*Chelydra serpentina*): chronic hypoxia alters autonomic regulation and gene expression. *Am. J. Physiol. Regul. Integr. Comp. Physiol.* 304, R966-R979. 10.1152/ajpregu.00595.2012 23552497

[JEB245530C55] Eme, J., Mueller, C., Manzon, R., Somers, C., Boreham, D. and Wilson, J. (2015). Critical windows in embryonic development: shifting incubation temperatures alter heart rate and oxygen consumption of Lake Whitefish (*Coregonus clupeaformis*) embryos and hatchlings. *Comp. Biochem. Physiol. Part A: Mol. Integr. Physiol.* 179, 71-80. 10.1016/j.cbpa.2014.09.005 25236178

[JEB245530C56] Eme, J., Tate, K. B., Rhen, T. and Crossley, D. A. 2nd. (2021). Cardiovascular responses to putative chemoreceptor stimulation of embryonic common snapping turtles (*Chelydra serpentina*) chronically incubated in hypoxia (10% O(2)). *Comp. Biochem. Physiol. A Mol. Integr. Physiol.* 259, 110977. 10.1016/j.cbpa.2021.110977 33984502

[JEB245530C57] Esbaugh, A. J. (2018). Physiological implications of ocean acidification for marine fish: emerging patterns and new insights. *J. Comp. Physiol. B* 188, 1-13. 10.1007/s00360-017-1105-6 28547292

[JEB245530C58] Everaert, N., Kamers, B., Witters, A., De Smit, L., Debonne, M., Decuypere, E. and Bruggeman, V. (2007). Effect of four percent carbon dioxide during the second half of incubation on embryonic development, hatching parameters, and posthatch growth. *Poult. Sci.* 86, 1372-1379. 10.1093/ps/86.7.1372 17575184

[JEB245530C59] Fares, W., Shahein, E., Rizk, R. and El-Hanoun, A. (2012). Carbon dioxide as affected by ventilation process during early stage of incubation and its relation with embryonic development, hormone levels, hatching parameters and post-hatch chicks growth. *Egypt. Poult. Sci. J.* 32, 23-41.

[JEB245530C60] Fritsche, R. and Burggren, W. (1996). Development of cardiovascular responses to hypoxia in larvae of the frog *Xenopus laevis*. *Am. J. Physiol.Regul. Integr. Comp. Physiol.* 271, R912-R917. 10.1152/ajpregu.1996.271.4.R912 8897981

[JEB245530C61] Galli, G. L., Crossley, J., Elsey, R. M., Dzialowski, E. M., Shiels, H. A. and Crossley, D. A. 2nd. (2016). Developmental plasticity of mitochondrial function in American alligators, *Alligator mississippiensis*. *Am. J. Physiol. Regul. Integr. Comp. Physiol.* 311, R1164-R1172. 10.1152/ajpregu.00107.2016 27707718 PMC5256979

[JEB245530C62] Galli, G. L. J., Ruhr, I. M., Crossley, J. and Crossley, D. A. (2021). The long-term effects of developmental hypoxia on cardiac mitochondrial function in snapping turtles. *Front. Physiol.* 12, 689684. 10.3389/fphys.2021.689684 34262478 PMC8273549

[JEB245530C63] Galli, G. L. J., Lock, M. C., Smith, K. L. M., Giussani, D. A. and Crossley, D. A. 2nd. (2023). Effects of developmental hypoxia on the vertebrate cardiovascular system. *Physiology (Bethesda)* 38, 53-62. 10.1152/physiol.00022.2022 36317939

[JEB245530C64] Giussani, D. A. (2016). The fetal brain sparing response to hypoxia: physiological mechanisms. *J. Physiol.* 594, 1215-1230. 10.1113/JP271099 26496004 PMC4721497

[JEB245530C65] Giussani, D. A. (2021). Breath of life: heart disease link to developmental hypoxia. *Circulation* 144, 1429-1443. 10.1161/CIRCULATIONAHA.121.054689 34694887 PMC8542082

[JEB245530C66] Giussani, D. A., Phillips, P. S., Anstee, S. and Barker, D. J. (2001). Effects of altitude versus economic status on birth weight and body shape at birth. *Pediatr. Res.* 49, 490-494. 10.1203/00006450-200104000-00009 11264431

[JEB245530C67] Giussani, D. A., Salinas, C. E., Villena, M. and Blanco, C. E. (2007). The role of oxygen in prenatal growth: studies in the chick embryo. *J. Physiol.* 585, 911-917. 10.1113/jphysiol.2007.141572 17962335 PMC2375513

[JEB245530C68] Golub, M., Koupaei-Abyazani, N., Vesala, T., Mammarella, I., Ojala, A., Bohrer, G., Weyhenmeyer, G., Blanken, P. D., Eugster, W. and Koebsch, F. et al. (2023). Diel, Seasonal, and Inter-annual variation in carbon dioxide effluxes from lakes and reservoirs. *Environ. Res. Lett.* 18, 034046. 10.1088/1748-9326/acb834

[JEB245530C69] Gómez, A. and Lunt, D. H. (2007). Refugia within refugia: patterns of phylogeographic concordance in the Iberian peninsula. In *Phylogeography of Southern European Refugia: Evolutionary perspectives on the origins and conservation of European biodiversity* (ed. S. Weiss and N. Ferrand), pp. 155-188. Dordrecht: Springer Netherlands.

[JEB245530C70] Grigg, G. C., Wells, R. M. G. and Beard, L. A. (1993). Allosteric control of oxygen binding by haemoglobin during embryonic development in the crocodile *Crocodylus porosus*: the role of red cell organic phosphates and carbon dioxide. *J. Exp. Biol.* 175, 15-32. 10.1242/jeb.175.1.15

[JEB245530C71] Grillitsch, S., Medgyesy, N., Schwerte, T. and Pelster, B. (2005). The influence of environmental P O2 on hemoglobin oxygen saturation in developing zebrafish *Danio rerio*. *J. Exp. Biol.* 208, 309-316. 10.1242/jeb.01410 15634850

[JEB245530C72] Henson, S. A., Beaulieu, C., Ilyina, T., John, J. G., Long, M., Séférian, R., Tjiputra, J. and Sarmiento, J. L. (2017). Rapid emergence of climate change in environmental drivers of marine ecosystems. *Nat. Commun.* 8, 14682. 10.1038/ncomms14682 28267144 PMC5343512

[JEB245530C73] Herrera, E. A., Salinas, C., Blanco, C., Villena, M. and Giussani, D. A. (2013). High altitude hypoxia and blood pressure dysregulation in adult chickens. *J. Dev. Orig. Health Dis.* 4, 69-76. 10.1017/S204017441200058X 25080183

[JEB245530C74] Ishimatsu, A., Hayashi, M. and Kikkawa, T. (2008). Fishes in high-CO_2_, acidified oceans. *Mar. Ecol. Prog. Ser.* 373, 295-302. 10.3354/meps07823

[JEB245530C75] Itani, N., Skeffington, K. L., Beck, C., Niu, Y. and Giussani, D. A. (2016). Melatonin rescues cardiovascular dysfunction during hypoxic development in the chick embryo. *J. Pineal Res.* 60, 16-26. 10.1111/jpi.12283 26444711 PMC4832387

[JEB245530C76] Itani, N., Salinas, C. E., Villena, M., Skeffington, K. L., Beck, C., Villamor, E., Blanco, C. E. and Giussani, D. A. (2018). The highs and lows of programmed cardiovascular disease by developmental hypoxia: studies in the chicken embryo. *J. Physiol.* 596, 2991-3006. 10.1113/JP274111 28983923 PMC6068112

[JEB245530C77] Itani, N., Skeffington, K. L., Beck, C., Niu, Y., Katzilieris-Petras, G., Smith, N. and Giussani, D. A. (2020). Protective effects of pravastatin on the embryonic cardiovascular system during hypoxic development. *FASEB J.* 34, 16504-16515. 10.1096/fj.202001743R 33094855

[JEB245530C78] Iyer, N. V., Kotch, L. E., Agani, F., Leung, S. W., Laughner, E., Wenger, R. H., Gassmann, M., Gearhart, J. D., Lawler, A. M. and Aimee, Y. Y. et al. (1998). Cellular and developmental control of O_2_ homeostasis by hypoxia-inducible factor 1α. *Genes Dev.* 12, 149-162. 10.1101/gad.12.2.149 9436976 PMC316445

[JEB245530C79] Jackson, D. C. (2002). Hibernating without oxygen: physiological adaptations of the painted turtle. *J. Physiol.* 543, 731-737. 10.1113/jphysiol.2002.024729 12231634 PMC2290531

[JEB245530C80] Jacob, E., Drexel, M., Schwerte, T. and Pelster, B. (2002). Influence of hypoxia and of hypoxemia on the development of cardiac activity in zebrafish larvae. *Am. J. Physiol. Regul. Integr. Comp. Physiol.* 283, R911-R917. 10.1152/ajpregu.00673.2001 12228061

[JEB245530C81] Johnston, E. F., Alderman, S. L. and Gillis, T. E. (2013). Chronic hypoxia exposure of trout embryos alters swimming performance and cardiac gene expression in larvae. *Physiol. Biochem. Zool.* 86, 567-575. 10.1086/672012 23995487

[JEB245530C82] Jonker, S. S., Giraud, G. D., Espinoza, H. M., Davis, E. N. and Crossley, D. A. 2nd. (2015). Effects of chronic hypoxia on cardiac function measured by pressure-volume catheter in fetal chickens. *Am. J. Physiol. Regul. Integr. Comp. Physiol.* 308, R680-R689. 10.1152/ajpregu.00484.2014 25652537 PMC4422373

[JEB245530C83] Jonz, M. G. and Nurse, C. A. (2005). Development of oxygen sensing in the gills of zebrafish. *J. Exp. Biol.* 208, 1537-1549. 10.1242/jeb.01564 15802677

[JEB245530C84] Joyce, W., Miller, T. E., Elsey, R. M., Wang, T. and Crossley, D. A. 2nd. (2018). The effects of embryonic hypoxic programming on cardiovascular function and autonomic regulation in the American alligator (*Alligator mississippiensis*) at rest and during swimming. *J. Comp. Physiol. B Biochem. Syst. Eenviron. Physiol.* 188, 967-976. 10.1007/s00360-018-1181-2 30218146

[JEB245530C85] Kajimura, M., Croke, S. J., Glover, C. N. and Wood, C. M. (2004). Dogmas and controversies in the handling of nitrogenous wastes: the effect of feeding and fasting on the excretion of ammonia, urea and other nitrogenous waste products in rainbow trout. *J. Exp. Biol.* 207, 1993-2002. 10.1242/jeb.00901 15143133

[JEB245530C86] Kajimura, S., Aida, K. and Duan, C. (2005). Insulin-like growth factor-binding protein-1 (IGFBP-1) mediates hypoxia-induced embryonic growth and developmental retardation. *Proc. Natl. Acad. Sci. USA* 102, 1240-1245. 10.1073/pnas.0407443102 15644436 PMC545835

[JEB245530C87] Kam, Y. C. (1993). Physiological effects of hypoxia on metabolism and growth of turtle embryos. *Respir. Physiol.* 92, 127-138. 10.1016/0034-5687(93)90033-7 8327786

[JEB245530C88] Kamei, H., Ding, Y., Kajimura, S., Wells, M., Chiang, P. and Duan, C. (2011). Role of IGF signaling in catch-up growth and accelerated temporal development in zebrafish embryos in response to oxygen availability. *Development* 138, 777-786. 10.1242/dev.056853 21266413

[JEB245530C89] Kamei, H., Yoneyama, Y., Hakuno, F., Sawada, R., Shimizu, T., Duan, C. and Takahashi, S.-I. (2018). Catch-up growth in zebrafish embryo requires neural crest cells sustained by Irs1 signaling. *Endocrinology* 159, 1547-1560. 10.1210/en.2017-00847 29390112

[JEB245530C90] Kang, J., Ma, X. and He, S. (2017). Evidence of high-altitude adaptation in the glyptosternoid fish, *Creteuchiloglanis macropterus* from the Nujiang River obtained through transcriptome analysis. *BMC Evol. Biol.* 17, 229. 10.1186/s12862-017-1074-0 29169322 PMC5701497

[JEB245530C91] Lei, Y., Yang, L., Zhou, Y., Wang, C., Lv, W., Li, L. and He, S. (2021). Hb adaptation to hypoxia in high-altitude fishes: fresh evidence from schizothoracinae fishes in the Qinghai-Tibetan Plateau. *Int. J. Biol. Macromol.* 185, 471-484. 10.1016/j.ijbiomac.2021.06.186 34214574

[JEB245530C92] Leo, E., Dahlke, F. T., Storch, D., Pörtner, H. O. and Mark, F. C. (2018). Impact of Ocean Acidification and Warming on the bioenergetics of developing eggs of Atlantic herring *Clupea harengus*. *Conserv. Physiol.* 6, coy050. 10.1093/conphys/coy050 30254749 PMC6142905

[JEB245530C93] León-Velarde, F. and Monge-C, C. (2004). Avian embryos in hypoxic environments. *Respir. Physiol. Neurobiol.* 141, 331-343. 10.1016/j.resp.2004.02.010 15288603

[JEB245530C94] Levesque, K. D., Wright, P. A. and Bernier, N. J. (2019). Cross talk without cross tolerance: effect of rearing temperature on the hypoxia response of embryonic zebrafish. *Physiol. Biochem. Zool.* 92, 349-364. 10.1086/703178 31070548

[JEB245530C95] Li, X., Wu, P., Ma, L., Huebner, C., Sun, B. and Li, S. (2020). Embryonic and post-embryonic responses to high-elevation hypoxia in a low-elevation lizard. *Integr. Zool.* 15, 338-348. 10.1111/1749-4877.12441 32297704

[JEB245530C96] Lindgren, I. and Altimiras, J. (2013). Prenatal hypoxia programs changes in β-adrenergic signaling and postnatal cardiac contractile dysfunction. *Am. J. Physiol. Regul. Integr. Comp. Physiol.* 305, R1093-R1101. 10.1152/ajpregu.00320.2013 24089370

[JEB245530C97] Lourens, A., van den Brand, H., Heetkamp, M. J. W., Meijerhof, R. and Kemp, B. (2007). Effects of eggshell temperature and oxygen concentration on embryo growth and metabolism during incubation. *Poult. Sci.* 86, 2194-2199. 10.1093/ps/86.10.2194 17878449

[JEB245530C98] Maksimov, V. and Korostyshevskaia, I. (2012). Morphogenesis and reaction to hypoxia of atrial myoendocrine cells in chick embryos (*Gallus gallus*). *Zh. Evol. Biokhim. Fiziol.* 48, 502-508. 23136760

[JEB245530C99] Mandic, M., Best, C. and Perry, S. F. (2020). Loss of hypoxia-inducible factor 1α affects hypoxia tolerance in larval and adult zebrafish (*Danio rerio*). *Proc. R. Soc. B* 287, 20200798. 10.1098/rspb.2020.0798 PMC7287360 32453991

[JEB245530C100] Mason, J. C. (1969). Hypoxial stress prior to emergence and competition among coho salmon fry. *J. Fish. Res. Board Can.* 26, 63-91. 10.1139/f69-007

[JEB245530C101] Melendez, C. L. and Mueller, C. A. (2021). Effect of increased embryonic temperature during developmental windows on survival, morphology and oxygen consumption of rainbow trout (*Oncorhynchus mykiss*). *Comp. Biochem. Physiol. Part A: Mol. Integr. Physiol.* 252, 110834. 10.1016/j.cbpa.2020.110834 33152473

[JEB245530C102] Mikloska, K. V., Zrini, Z. A. and Bernier, N. J. (2022). Severe hypoxia exposure inhibits larval brain development but does not affect the capacity to mount a cortisol stress response in zebrafish. *J. Exp. Biol.* 225, jeb243335. 10.1242/jeb.243335 34931659

[JEB245530C103] Miller, S. (2013). Cardiac responses to carbon dioxide in developing zebrafish (*Danio rerio*). MSc thesis, Université d'Ottawa/University of Ottawa.

[JEB245530C104] Miller, S. C., Reeb, S. E., Wright, P. A. and Gillis, T. E. (2008). Oxygen concentration in the water boundary layer next to rainbow trout (*Oncorhynchus mykiss*) embryos is influenced by hypoxia exposure time, metabolic rate, and water flow. *Can. J. Fish. Aquat. Sci.* 65, 2170-2177. 10.1139/F08-123

[JEB245530C105] Miller, S. C., Gillis, T. E. and Wright, P. A. (2011). The ontogeny of regulatory control of the rainbow trout (*Oncorhynchus mykiss*) heart and how this is influenced by chronic hypoxia exposure. *J. Exp. Biol.* 214, 2065-2072. 10.1242/jeb.054825 21613523

[JEB245530C106] Moore, F. B., Hosey, M. and Bagatto, B. (2006). Cardiovascular system in larval zebrafish responds to developmental hypoxia in a family specific manner. *Front. Zool.* 3, 4. 10.1186/1742-9994-3-4 16539736 PMC1479343

[JEB245530C107] Mortola, J. P., Wills, K., Trippenbach, T. and Al Awam, K. (2010). Interactive effects of temperature and hypoxia on heart rate and oxygen consumption of the 3-day old chicken embryo. *Comp. Biochem. Physiol. Part A: Mol. Integr. Physiol.* 155, 301-308. 10.1016/j.cbpa.2009.11.003 19914389

[JEB245530C108] Mu, J., Jin, F., Wang, J., Zheng, N. and Cong, Y. (2015). Effects of CO 2-driven ocean acidification on early life stages of marine medaka (*Oryzias melastigma*). *Biogeosciences* 12, 3861-3868. 10.5194/bg-12-3861-2015

[JEB245530C109] Mueller, C. A., Joss, J. M. and Seymour, R. S. (2011). The energy cost of embryonic development in fishes and amphibians, with emphasis on new data from the Australian lungfish, Neoceratodus forsteri. *J. Comp. Physiol. B Biochem. Syst. Eenviron. Physiol.* 181, 43-52. 10.1007/s00360-010-0501-y 20676654

[JEB245530C110] Mueller, C. A., Tazawa, H. and Burggren, W. W. (2017). Dynamics of acid-base and hematological regulation in day 15 chicken embryos (*Gallus gallus domesticus*) exposed to graded hypercapnia and hypoxia. *Respir. Physiol. Neurobiol.* 239, 55-63. 10.1016/j.resp.2017.02.001 28189709

[JEB245530C111] Murray, C. S., Fuiman, L. A. and Baumann, H. (2016). Consequences of elevated CO_2_ exposure across multiple life stages in a coastal forage fish. *ICES J. Mar. Sci.* 74, 1051-1061. 10.1093/icesjms/fsw179

[JEB245530C112] Neate-Clegg, M. H. and Tingley, M. W. (2023). Building a mechanistic understanding of climate-driven elevational shifts in birds. *PLOS Climate* 2, e0000174.

[JEB245530C113] Owerkowicz, T., Elsey, R. M. and Hicks, J. W. (2009). Atmospheric oxygen level affects growth trajectory, cardiopulmonary allometry and metabolic rate in the American alligator (*Alligator mississippiensis*). *J. Exp. Biol.* 212, 1237-1247. 10.1242/jeb.023945 19376944 PMC2726848

[JEB245530C114] Parker, S. L. and Dimkovikj, V. H. (2019). Effects of regional hypoxia and incubation temperature on growth, differentiation, heart mass, and oxygen consumption in embryos of the leopard gecko (*Eublepharis macularius*). *Comp. Biochem. Physiol. A Mol. Integr. Physiol.* 227, 51-59. 10.1016/j.cbpa.2018.09.006 30240787

[JEB245530C115] Pelster, B. (1999). Environmental influences on the development of the cardiac system in fish and amphibians. *Comp. Biochem. Physiol. A Mol. Integr. Physiol.* 124, 407-412. 10.1016/S1095-6433(99)00132-4 10682238

[JEB245530C116] Phillips, J. C., McKinley, G. A., Bennington, V., Bootsma, H. A., Pilcher, D. J., Sterner, R. W. and Urban, N. R. (2015). The potential for CO_2_-induced acidification in freshwater: a Great Lakes case study. *Oceanography* 28, 136-145. 10.5670/oceanog.2015.37

[JEB245530C117] Pörtner, H., Karl, D., Boyd, P., Cheung, W., Lluch-Cota, S., Nojiri, Y., Schmidt, D., Zavialov, P., Alheit, J. and Aristegui, J. (2014). *Ocean Systems. Climate change 2014: impacts, adaptation, and vulnerability. Part A: global and sectoral aspects. Contribution of working group II to the fifth assessment report of the Intergovernmental Panel on Climate Change*, pp. 411-484. Cambridge University Press.

[JEB245530C118] Pottier, P., Burke, S., Zhang, R. Y., Noble, D. W. A., Schwanz, L. E., Drobniak, S. M. and Nakagawa, S. (2022). Developmental plasticity in thermal tolerance: ontogenetic variation, persistence, and future directions. *Ecol. Lett.* 25, 2245-2268. 10.1111/ele.14083 36006770 PMC9804923

[JEB245530C119] Ragsdale, A., Ortega-Recalde, O., Dutoit, L., Besson, A. A., Chia, J. H. Z., King, T., Nakagawa, S., Hickey, A., Gemmell, N. J., Hore, T. et al. (2022). Paternal hypoxia exposure primes offspring for increased hypoxia resistance. *BMC Biol.* 20, 185. 10.1186/s12915-022-01389-x 36038899 PMC9426223

[JEB245530C177] Richards, J. G. (2011). Physiological, behavioral and biochemical adaptations of intertidal fishes to hypoxia. *J. Exp. Biol.* 214, 191-199. 21177940 10.1242/jeb.047951

[JEB245530C120] Robertson, C. E., Wright, P. A., Köblitz, L. and Bernier, N. J. (2014). Hypoxia-inducible factor-1 mediates adaptive developmental plasticity of hypoxia tolerance in zebrafish, Danio rerio. *Proc. Biol. Sci.* 281, 20140637. 10.1098/rspb.2014.0637 24850928 PMC4046416

[JEB245530C121] Rombough, P. J. (1988). Growth, aerobic metabolism, and dissolved oxygen requirements of embryos and alevins of steelhead, *Salmo gairdneri*. *Can. J. Zool.* 66, 651-660. 10.1139/z88-097

[JEB245530C122] Rouwet, E. V., Tintu, A. N., Schellings, M. W., van Bilsen, M., Lutgens, E., Hofstra, L., Slaaf, D. W., Ramsay, G. and Le Noble, F. A. (2002). Hypoxia induces aortic hypertrophic growth, left ventricular dysfunction, and sympathetic hyperinnervation of peripheral arteries in the chick embryo. *Circulation* 105, 2791-2796. 10.1161/01.CIR.0000017497.47084.06 12057996

[JEB245530C123] Rubenstein, M. A., Weiskopf, S. R., Bertrand, R., Carter, S. L., Comte, L., Eaton, M. J., Johnson, C. G., Lenoir, J., Lynch, A. J. and Miller, B. W. et al. (2023). Climate change and the global redistribution of biodiversity: substantial variation in empirical support for expected range shifts. *Environ. Evid.* 12, 1-21. 10.1186/s13750-023-00296-0 39294691 PMC11378804

[JEB245530C124] Ruhr, I. M., McCourty, H., Bajjig, A., Crossley, D. A., Shiels, H. A. and Galli, G. L. J. (2019). Developmental plasticity of cardiac anoxia-tolerance in juvenile common snapping turtles (*Chelydra serpentina*). *Proc. R. Soc. B* 286, 20191072. 10.1098/rspb.2019.1072 PMC6599983 31238852

[JEB245530C125] Ruhr, I., Bierstedt, J., Rhen, T., Das, D., Singh, S. K., Miller, S., Crossley, D. A. and Galli, G. L. J. (2021). Developmental programming of DNA methylation and gene expression patterns is associated with extreme cardiovascular tolerance to anoxia in the common snapping turtle. *Epigenetics Chromatin* 14, 42. 10.1186/s13072-021-00414-7 34488850 PMC8420019

[JEB245530C126] Ruijtenbeek, K., Le Noble, F., Janssen, G., Kessels, C., Fazzi, G., Blanco, C. and De Mey, J. (2000). Chronic hypoxia stimulates periarterial sympathetic nerve development in chicken embryo. *Circulation* 102, 2892-2897. 10.1161/01.CIR.102.23.2892 11104750

[JEB245530C127] Salinas, C., Blanco, C., Villena, M., Camm, E., Tuckett, J., Weerakkody, R., Kane, A., Shelley, A., Wooding, F. and Quy, M. et al. (2010). Cardiac and vascular disease prior to hatching in chick embryos incubated at high altitude. *J. Dev. Orig. Health Dis.* 1, 60-66. 10.1017/S2040174409990043 25142932

[JEB245530C128] Salinas, C. E., Villena, M., Blanco, C. E. and Giussani, D. A. (2011). Adrenocortical suppression in highland chick embryos is restored during incubation at sea level. *High Alt. Med. Biol.* 12, 79-87. 10.1089/ham.2010.1040 21452969

[JEB245530C129] Salinas, C. E., Blanco, C. E., Villena, M. and Giussani, D. A. (2014). High-altitude hypoxia and echocardiographic indices of pulmonary hypertension in male and female chickens at adulthood. *Circ. J.* 78, 1459-1464. 10.1253/circj.CJ-13-1329 24739224

[JEB245530C130] Sandovici, I., Fernandez-Twinn, D. S., Hufnagel, A., Constância, M. and Ozanne, S. E. (2022). Sex differences in the intergenerational inheritance of metabolic traits. *Nat. Metab.* 4, 507-523. 10.1038/s42255-022-00570-4 35637347

[JEB245530C131] Sartori, M. R., Kohl, Z. F., Taylor, E. W., Abe, A. S. and Crossley Ii, D. A. (2019). Blood flow distribution in embryonic common snapping turtles *Chelydra serpentina* (Reptilia; Chelonia) during acute hypoxia and α-adrenergic regulation. *Comp. Biochem. Physiol. A Mol. Integr. Physiol.* 238, 110575. 10.1016/j.cbpa.2019.110575 31505219

[JEB245530C132] Scheuffele, H. (2017). *Effects of Ocean Acidification on the phenotypic plasticity and functional properties of European sea bass (Dicentrarchus labrax) haemoglobin*. MSc thesis, Bremen University.

[JEB245530C133] Schwerte, T., Überbacher, D. and Pelster, B. (2003). Non-invasive imaging of blood cell concentration and blood distribution in hypoxic incubated zebrafish in vivo (*Danio rerio*). *J. Exp. Biol.* 206, 1299-1307. 10.1242/jeb.00249 12624165

[JEB245530C134] Seymour, R. S. and Ackerman, R. A. (1980). Adaptations to underground nesting in birds and reptiles. *Am. Zool.* 20, 437-447. 10.1093/icb/20.2.437

[JEB245530C135] Shang, E. H. and Wu, R. S. (2004). Aquatic hypoxia is a teratogen and affects fish embryonic development. *Environ. Sci. Technol.* 38, 4763-4767. 10.1021/es0496423 15487785

[JEB245530C136] Sharma, S. K., Lucitti, J. L., Nordman, C., Tinney, J. P., Tobita, K. and Keller, B. B. (2006). Impact of hypoxia on early chick embryo growth and cardiovascular function. *Pediatr. Res.* 59, 116-120. 10.1203/01.pdr.0000191579.63339.90 16327005

[JEB245530C137] Shine, R. (2015). The evolution of oviparity in squamate reptiles: an adaptationist perspective. *J. Exp. Zool. Part B: Mol. Dev. Evol.* 324, 487-492. 10.1002/jez.b.22622 26036339

[JEB245530C138] Singh, S., Das, D. and Rhen, T. (2020). Embryonic temperature programs phenotype in reptiles. *Front. Physiol.* 11, 35. 10.3389/fphys.2020.00035 32082193 PMC7005678

[JEB245530C139] Skeffington, K. L., Beck, C., Itani, N., Niu, Y., Shaw, C. J. and Giussani, D. A. (2020). Hypertension programmed in adult hens by isolated effects of developmental hypoxia In Ovo. *Hypertension* 76, 533-544. 10.1161/HYPERTENSIONAHA.120.15045 32536277 PMC7340221

[JEB245530C140] Smith, B., Crossley, J. L., Elsey, R. M., Hicks, J. W. and Crossley, D. A. 2nd. (2019). Embryonic developmental oxygen preconditions cardiovascular functional response to acute hypoxic exposure and maximal β-adrenergic stimulation of anesthetized juvenile American alligators (*Alligator mississippiensis*). *J. Exp. Biol.* 222, jeb205419. 10.1242/jeb.205419 31548289

[JEB245530C141] Smith, B., Crossley, J. L., Conner, J., Elsey, R. M., Wang, T. and Crossley, D. A. (2023). Exposure to hypoxia during embryonic development affects blood flow patterns and heart rate in juvenile American alligators during digestion. *Comp. Biochem. Physiol. Part A: Mol. Integr. Physiol.* 282, 111440. 10.1016/j.cbpa.2023.111440 37169243

[JEB245530C142] Snyder, G. K., Byers, R. L. and Kayar, S. R. (1984). Effects of hypoxia on tissue capillarity in geese. *Respir. Physiol.* 58, 151-160. 10.1016/0034-5687(84)90144-0 6522870

[JEB245530C143] Souchet, J., Bossu, C., Darnet, E., Le Chevalier, H., Poignet, M., Trochet, A., Bertrand, R., Calvez, O., Martinez-Silvestre, A., Mossoll-Torres, M. et al. (2020a). High temperatures limit developmental resilience to high-elevation hypoxia in the snake *Natrix maura* (Squamata: Colubridae). *Biol. J. Linn. Soc.* 132, 116-133. 10.1093/biolinnean/blaa182

[JEB245530C144] Souchet, J., Gangloff, E. J., Micheli, G., Bossu, C., Trochet, A., Bertand, R., Clobert, J., Calvez, O., Martinez-Silbestre, A., Darnet, E. et al. (2020b). High-elevation hypoxia impacts perinatal physiology and performance in a potential montane colonizer. *Integr. Zool.* 15, 544-557. 10.1111/1749-4877.12468 32649806 PMC7689776

[JEB245530C145] Steele, S. L., Lo, K. H. A., Li, V. W. T., Cheng, S. H., Ekker, M. and Perry, S. F. (2009). Loss of M2 muscarinic receptor function inhibits development of hypoxic bradycardia and alters cardiac β-adrenergic sensitivity in larval zebrafish (*Danio rerio*). *Am. J. Physiol. Regul. Integr. Comp. Physiol.* 297, R412-R420. 10.1152/ajpregu.00036.2009 19515979

[JEB245530C146] Steele, S. L., Ekker, M. and Perry, S. F. (2011). Interactive effects of development and hypoxia on catecholamine synthesis and cardiac function in zebrafish (*Danio rerio*). *J. Comp. Physiol. B* 181, 527-538. 10.1007/s00360-010-0544-0 21197535

[JEB245530C147] Sultan, S. E. (2017). Developmental plasticity: re-conceiving the genotype. *Interface Focus* 7, 20170009. 10.1098/rsfs.2017.0009 28839928 PMC5566816

[JEB245530C148] Sun, C.-F., Tao, Y., Jiang, X.-Y. and Zou, S.-M. (2011). IGF binding protein 1 is correlated with hypoxia-induced growth reduce and developmental defects in grass carp (*Ctenopharyngodon idellus*) embryos. *Gen. Comp. Endocrinol.* 172, 409-415. 10.1016/j.ygcen.2011.04.005 21501614

[JEB245530C149] Sun, L., Ruan, J., Lu, M., Chen, M., Dai, Z. and Zuo, Z. (2019). Combined effects of ocean acidification and crude oil pollution on tissue damage and lipid metabolism in embryo–larval development of marine medaka (*Oryzias melastigma*). *Environ. Geochem. Health* 41, 1847-1860. 10.1007/s10653-018-0159-z 30066097

[JEB245530C150] Takeshita, R., Bursian, S. J., Colegrove, K. M., Collier, T. K., Deak, K., Dean, K. M., De Guise, S., DiPinto, L. M., Elferink, C. J., Esbaugh, A. J. et al. (2021). A review of the toxicology of oil in vertebrates: what we have learned following the Deepwater Horizon oil spill. *J. Toxicol. Environ. Health B Crit. Rev.* 24, 355-394. 10.1080/10937404.2021.1975182 34542016 PMC12404339

[JEB245530C151] Tate, K. B., Kohl, Z. F., Eme, J., Rhen, T. and Crossley, D. A. 2nd. (2015). Critical windows of cardiovascular susceptibility to developmental hypoxia in common snapping turtle (*Chelydra serpentina*) embryos. *Physiol. Biochem. Zool.* 88, 103-115. 10.1086/677683 25730266

[JEB245530C152] Tate, K. B., Rhen, T., Eme, J., Kohl, Z. F., Crossley, J., Elsey, R. M. and Crossley, D. A. 2nd. (2016). Periods of cardiovascular susceptibility to hypoxia in embryonic American alligators (*Alligator mississippiensis*). *Am. J. Physiol. Regul. Integr. Comp. Physiol.* 310, R1267-R1278. 10.1152/ajpregu.00320.2015 27101296 PMC4935500

[JEB245530C153] Tazawa, H. (1981). Effect of O2 and CO2 in N2, He, and SF6 on chick embryo blood pressure and heart rate. *J. Appl. Physiol.* 51, 1017-1022. 10.1152/jappl.1981.51.4.1017 6795162

[JEB245530C154] Tintu, A., Rouwet, E., Verlohren, S., Brinkmann, J., Ahmad, S., Crispi, F., van Bilsen, M., Carmeliet, P., Staff, A. C., Tjwa, M. et al. (2009). Hypoxia induces dilated cardiomyopathy in the chick embryo: mechanism, intervention, and long-term consequences. *PLoS One* 4, e5155. 10.1371/journal.pone.0005155 19357774 PMC2663815

[JEB245530C155] Ton, C., Stamatiou, D., Dzau, V. J. and Liew, C.-C. (2002). Construction of a zebrafish cDNA microarray: gene expression profiling of the zebrafish during development. *Biochem. Biophys. Res. Commun.* 296, 1134-1142. 10.1016/S0006-291X(02)02010-7 12207891

[JEB245530C156] Ton, C., Stamatiou, D. and Liew, C.-C. (2003). Gene expression profile of zebrafish exposed to hypoxia during development. *Physiol. Genomics* 13, 97-106. 10.1152/physiolgenomics.00128.2002 12700360

[JEB245530C157] Tong, C., Fei, T., Zhang, C. and Zhao, K. (2017). Comprehensive transcriptomic analysis of Tibetan Schizothoracinae fish *Gymnocypris przewalskii* reveals how it adapts to a high altitude aquatic life. *BMC Evol. Biol.* 17, 1-11. 10.1186/s12862-017-0925-z 28274203 PMC5343388

[JEB245530C158] Vagner, M., Zambonino-Infante, J.-L. and Mazurais, D. (2019). Fish facing global change: are early stages the lifeline? *Mar. Environ. Res.* 147, 159-178. 10.1016/j.marenvres.2019.04.005 31027942

[JEB245530C159] van Vliet, M. T. H., Thorslund, J., Strokal, M., Hofstra, N., Flörke, M., Ehalt Macedo, H., Nkwasa, A., Tang, T., Kaushal, S. S., Kumar, R. et al. (2023). Global river water quality under climate change and hydroclimatic extremes. *Nat. Rev. Earth Environ.* 4, 687-702. 10.1038/s43017-023-00472-3

[JEB245530C160] Vanderplancke, G., Claireaux, G., Quazuguel, P., Madec, L., Ferraresso, S., Sévère, A., Zambonino-Infante, J.-L. and Mazurais, D. (2015). Hypoxic episode during the larval period has long-term effects on European sea bass juveniles (*Dicentrarchus labrax*). *Mar. Biol.* 162, 367-376. 10.1007/s00227-014-2601-9

[JEB245530C161] Verhoelst, E., Ketelaere, B. D., Decuypere, E. and Baerdemaeker, J. D. (2011). The effect of early prenatal hypercapnia on the vascular network in the chorioallantoic membrane of the chicken embryo. *Biotechnol. Prog.* 27, 562-570. 10.1002/btpr.569 21365785

[JEB245530C162] Villalobos, C., Love, B. A. and Olson, M. B. (2020). Ocean acidification and ocean warming effects on pacific herring (*Clupea pallasi*) early life stages. *Front. Mar. Sci.* 7, 597899. 10.3389/fmars.2020.597899

[JEB245530C163] Villamor, E., Kessels, C. G., Ruijtenbeek, K., van Suylen, R. J., Belik, J., de Mey, J. G. and Blanco, C. E. (2004). Chronic in ovo hypoxia decreases pulmonary arterial contractile reactivity and induces biventricular cardiac enlargement in the chicken embryo. *Am. J. Physiol. Regul. Integr. Comp. Physiol.* 287, R642-R651. 10.1152/ajpregu.00611.2003 15117730

[JEB245530C164] Wang, G. L. and Semenza, G. L. (1996). Molecular basis of hypoxia-induced erythropoietin expression. *Curr. Opin Hematol.* 3, 156-162. 10.1097/00062752-199603020-00009 9372067

[JEB245530C165] Warburton, S. J., Hastings, D. and Wang, T. (1995). Responses to chronic hypoxia in embryonic alligators. *J. Exp. Zool.* 273, 44-50. 10.1002/jez.1402730106 7561723

[JEB245530C166] Wearing, O., Eme, J., Kemp, A. and Crossley II, D. (2014). Impact of hypercapnic incubation on hatchling common snapping turtle (*Chelydra serpentina*) growth and metabolism. *FASEB J.* 28, 1101.3. 10.1096/fasebj.28.1_supplement.1101.3

[JEB245530C167] Wearing, O. H., Conner, J., Nelson, D., Crossley, J. and Crossley, D. A. 2nd. (2017). Embryonic hypoxia programmes postprandial cardiovascular function in adult common snapping turtles (*Chelydra serpentina*). *J. Exp. Biol.* 220, 2589-2597. 10.1242/jeb.160549 28495871

[JEB245530C168] Wearing, O. H., Eme, J., Rhen, T. and Crossley, D. A. 2nd. (2016). Phenotypic plasticity in the common snapping turtle (*Chelydra serpentina*): long-term physiological effects of chronic hypoxia during embryonic development. *Am. J. Physiol. Regul. Integr. Comp. Physiol.* 310, R176-R184. 10.1152/ajpregu.00293.2015 26608655

[JEB245530C169] Whitehouse, L. M. and Manzon, R. G. (2019). Hypoxia alters the expression of hif-1a mRNA and downstream HIF-1 response genes in embryonic and larval lake whitefish (*Coregonus clupeaformis*). *Comp. Biochem. Physiol. Part A: Mol. Integr. Physiol.* 230, 81-90. 10.1016/j.cbpa.2019.01.005 30659950

[JEB245530C170] Wood, A. T., Clark, T. D., Andrewartha, S. J., Elliott, N. G. and Frappell, P. B. (2017). Developmental hypoxia has negligible effects on long-term hypoxia tolerance and aerobic metabolism of Atlantic salmon (*Salmo salar*). *Physiol. Biochem. Zool.* 90, 494-501. 10.1086/692250 28459654

[JEB245530C171] Wood, A. T., Andrewartha, S. J., Elliott, N. G., Frappell, P. B. and Clark, T. D. (2019a). Hypoxia during incubation does not affect aerobic performance or haematology of Atlantic salmon (*Salmo salar*) when re-exposed in later life. *Conserv. Physiol.* 7, coz088. 10.1093/conphys/coz088 31798884 PMC6880253

[JEB245530C172] Wood, A. T., Clark, T. D., Elliott, N. G., Frappell, P. B. and Andrewartha, S. J. (2019b). Physiological effects of dissolved oxygen are stage-specific in incubating Atlantic salmon (*Salmo salar*). *J. Comp. Physiol. B* 189, 109-120. 10.1007/s00360-018-1199-5 30603847

[JEB245530C173] Woolway, R. I., Sharma, S. and Smol, J. P. (2022). Lakes in hot water: the impacts of a changing climate on aquatic ecosystems. *Bioscience* 72, 1050-1061. 10.1093/biosci/biac052 36325103 PMC9618276

[JEB245530C178] Wu, R. S. S. (2009). Effects of Hypoxia on Fish Reproduction and Development. Chapter 3. In *Fish Physiology*, vol. 27 (ed. J. G. Richards, A. P. Farrell and C. J. Brauner), pp. 79-141. Academic Press.

[JEB245530C174] Yaqoob, N. and Schwerte, T. (2010). Cardiovascular and respiratory developmental plasticity under oxygen depleted environment and in genetically hypoxic zebrafish (*Danio rerio*). *Comp. Biochem. Physiol. Part A: Mol. Integr. Physiol.* 156, 475-484. 10.1016/j.cbpa.2010.03.033 20363352

[JEB245530C175] Zambonino-Infante, J. L., Claireaux, G., Ernande, B., Jolivet, A., Quazuguel, P., Sévère, A., Huelvan, C. and Mazurais, D. (2013). Hypoxia tolerance of common sole juveniles depends on dietary regime and temperature at the larval stage: evidence for environmental conditioning. *Proc. R. Soc. B* 280, 20123022. 10.1098/rspb.2012.3022 PMC3619455 23486433

