# Supplementary material for: Developmental plasticity of the cardiovascular system in oviparous vertebrates: effects of chronic hypoxia and interactive stressors in the context of climate change
Source: J Exp Biol. 2024 Aug 7;227(20):jeb245530. doi: 10.1242/jeb.245530 (PMC11418206; doi:10.1242/jeb.245530)
Supplement: Supplementary information [file jexbio-227-245530-s1.pdf]

Table S1. Effects of chronic developmental hypoxia on body mass and cardiovascular parameters in avians, reptiles and fish

AVIANS	Stage	Body Temp (°C)	$[O_2]$ Control	$[O_2]$ Hypoxia	(A) Body mass		(B) Heart mass		(C) Heart-body weight ratio	(D) Heart rate		(E) O_2 carrying capacity	(F) Mito capacity		(G) Cardiac Fibrosis		(H) Sympathetic activity	
					Emb	Juv or Adult	Emb	Juv or Adult	Emb	Emb	Juv or Adult	Emb	Emb	Juv or Adult	Emb	Juv or Adult	Emb	Juv or Adult
Gallus gallus domesticus (leghorn): 20-21 days	1-6 doi	37.5	21%O_2	15%O_2	↓ [1]<br>— [2]		— [1,2]	— [1]				— [1]						
	6-12 doi	37.5	21%O_2	15%O_2	↓ [1]		↑ [1]	— [1]				↑ [1]						
	6-19 doi	38	21%O_2	15%O_2	↓ [3-7]	— [4]	↑ [3]<br>— [5]<br>↓ [6]		↑ [3, 5]<br>— [6]				↓ [6]				↑ [7]	
	7-14	37.5	21%O_2	15%O_2	↓ [8]							↑ [8]						
	12-18 doi	37.5	21%O_2	15%O_2	↓ [1]		— [1]	— [1]				— [1]						
	16-18 doi	37.5	21%O_2	15%O_2	↓ [9]		↓ [9]											
	0-19 & 1-20 doi	37.5	21%O_2	15%O_2	↓ [10-16]		↓ [10,15]		— [10]<br>↑ [11,13]	— [14,16]		↑ [12,15]				↑ [15]		
	0-21 doi	37.5	21% O_2	14% O_2	↓ [17,18]		↓ [17,18]					↑ [17,18]						
	1-21 doi	38	21%O_2	HB: 13%O_2	↓ [19]												↑ [19]	
	0-20 doi	38	21% O_2	HB: 13%O_2	↓ [20]							↑ [20]						

Journal of Experimental Biology • Supplementary information

AVIANS	Stage	Body Temp (°C)	$[O_2]$ Control	$[O_2]$ Hypoxia	(A) Body mass		(B) Heart mass		(C) Heart-body weight ratio	(D) Heart rate		(E) O_2 carrying capacity	(F) Mito capacity		(G) Cardiac Fibrosis		(H) Sympathetic activity	
					Emb	Juv or Adult	Emb	Juv or Adult	Emb	Emb	Juv or Adult	Emb	Emb	Juv or Adult	Emb	Juv or Adult	Emb	Juv or Adult
**Gallus gallus domesticus (bovans brown): 20-21 days**	1-21 doi	37.9	21%O_2	14%O_2	↓ [21-23]	↓ [23]	↑ [21]		— [21]			↑ [22,23]	↓ [22]					↑ [23]
**Gallus gallus domesticus (broiler): 20-21 days**	1-21 or 0-19 doi	37.8	21%O_2	14%O_2	↓ [24-26]	— [24] ↓ [25]	— [25]	— [25]	↑ [24]	— [26]						— [25]	↑ [24,26]	↓ [24] ↑ [25]
	1-20 doi	37.8	21%O_2	15%O_2	↓ [10]		↓ [10]		↓ [10]									
	6-19 doi	37.8	21%O_2	15%O_2	↓ [4]	↓ [4]	↑ [4]											
	9-19	37.8	21%O_2	17%O_2	— [27]		— [27]											
		38.9	21%O_2	17%O_2	— [27]		— [27]											
**Gallus gallus (red junglefowl): 19-21 days**	1-20 doi	37.8	21%O_2	15%O_2	↓ [10]		↓ [10]		↓ [10]									
***Branta canadensis* (Canada goose) 28 days**	0-28	37	16%O_2	12%O_2	— [28,29]							↑ [29]						
***Anser indicus* (bar-headed goose): 27-30 days**	0-28	37	16%O_2	12%O_2	— [28]													

REPTILES	Stage	Body Temp (°C)	$[O_2]$ Control	$[O_2]$ Hypoxia	(A) Body mass		(B) Heart mass		(C) Heart-body weight ratio	(D) Heart rate		(E) O_2 carrying capacity	(F) Mito capacity		(G) Cardiac Fibrosis		(H) Sympathetic activity	
					Emb	Juv or Adult	Emb	Juv or Adult	Emb	Emb	Juv or Adult	Emb	Emb	Juv or Adult	Emb	Juv or Adult	Emb	Juv or Adult
American alligator (*Alligator mississippiensis*): 63-68 days	0-90%	30	21%O_2	10%O_2	↓ 30-34	— 35-37 ↓ 38,39	— 30,31		↑ 30,31	↓ 30,33,34	— 35	↑ 40	— 38	↑ 38				
	0-80%	30	21%O_2	10%O_2	↓ 30,34		— 30		↑ 30	↓ 34 — 30								
	0-70%	30	21%O_2	10%O_2	— 30 ↓ 33,34		— 30		— 30	— 30 ↓ 33,34							↑ 34	
	0-60%	30	21%O_2	15%O_2	— 30		— 30		— 30	— 30								
	0-90%	30	21%O_2	15%O_2	— 30		— 30		— 30	— 30								
	0-80%	30	21%O_2	15%O_2	— 30		— 30		— 30	— 30								
	0-70%	30	21%O_2	15%O_2	— 30		— 30		— 30	— 30								
	0-60%	30	21%O_2	15%O_2	— 30		— 30		— 30	— 30								
Snapping turtle (Chelydra serpentina): 80-90 days	0-90%	30	21%O_2	10%O_2	↓ 41-43	↓ 44 — 45–47	↑ 43		↑ 41-43,48	— 41 ↑ 42		↑ 49		↑ 47			↑ 42	
	0-70%	30	21%O_2	10%O_2	↓ 41-43		— 43		↑ 41,42,48 — 43	— 41,42				↑ 38			↑ 42	

Journal of Experimental Biology • Supplementary information

REPTILES	Stage	Body Temp (°C)	$[O_2]$ Control	$[O_2]$ Hypoxia	(A) Body mass		(B) Heart mass		(C) Heart-body weight ratio	(D) Heart rate		(E) O_2 carrying capacity	(F) Mito capacity		(G) Cardiac Fibrosis		(H) Sympathetic activity	
					Emb	Juv or Adult	Emb	Juv or Adult	Emb	Emb	Juv or Adult	Emb	Emb	Juv or Adult	Emb	Juv or Adult	Emb	Juv or Adult
Florida red-bellied turtle (Pseudemys Nelson): 45-80 days	0-90%	30	$21\%O_2$	$10\%O_2$	↓ [49]		— [49]		↑ [49]			↑ [49]						
Leopard gecko (Eublepharis macularius): 45-53 days	0-70%	34	$21\%O_2$	Regional hypoxia	↓ [50]		↓ [50]		↑ [50]					↑ [38]				
	0-70%	28	$21\%O_2$	Regional hypoxia	↓ [50]		↓ [50]		↑ [50]									
Banded red snake (Lycodon rufozonatu): 50 days	10-100%	28	$21\%O_2$	Regional hypoxia	— [51]				↑ [51]									
Chinese softshell turtle (Pelodiscus sinensis): 60 days	0-100%	28	$21\%O_2$	Regional hypoxia	— [51]				— [51]									
Common wall lizard (Podarcis muralis): 42-77 days	0-100%	24	$21\%O_2$	HB $15\%O_2$	↓ [52]<br>— [53]		↑ [52]			↓ [52,53]								
	0-100%	24	$21\%O_2$	HB $17\%O_2$	— [53]					↓ [53]								

**Viperine snake (*Natrix maura*)**	0-100%	24	21%O_2	HB 15%O_2	— [54]					— [54]								
	0-100%	28	21%O_2	HB 15%O_2	↓ [55]					↑ [55]								
	0-100%	32	21%O_2	HB 15%O_2	↓ [54]					↓ [54]								
**Class and species**	**Stage**	**Body Temp (°C)**	**[O_2] Control**	**[O_2] Hypoxia**	**(A) Body mass**		**(B) Heart mass**		**(C) Heart-body weight ratio**	**(D) Heart rate**		**(E) O_2 carrying capacity**	**(F) Mito capacity**		**(G) Cardiac Fibrosis**		**(H) Sympathetic activity**	
**FISH**					**Emb**	**Juv or Adult**	**Emb**	**Juv or Adult**	**Emb**	**Emb**	**Juv or Adult**	**Emb**	**Emb**	**Juv or Adult**	**Emb**	**Juv or Adult**	**Emb**	**Juv or Adult**
**Zebrafish (*Danio rerio*): 3-4 days**	0-2 dpf	25	6.5 mg/L	0.8 mg/L						↓ [56]								
	1-5 dpf	25	7.5 mg/L	3.8 mg/L	— [57]		↑ [57]			↑ [57]								
	0-1 dpf	28	7.5 mg /L	4.3 mg /L	— [58]	↓ [58]				— [58]								
	0-1 dpf	28	6.5 mg/L	0.6 mg /L	↓ [59]													
	2-4 dpf	28	100%	5%									↓ [60]					
	0-4 dpf	28	6 mg/L	1-2 mg/L			↓ [61,62]			↓ [61,62]								
	1-15 dpf	28	7.5 mg/L	3.3 mg/L	↓ [63]							↑ [63]						
	1-5 dpf	28	7.5 mg/L	3.8 mg/L	— [57]		↑ [57]			↑ [57]								
	5-9 dpf	28	7.5 mg/L	1.5 mg/L						↓ [64]							↑ [64]	
	1-5 dpf	28	7.5 mg/L	1.5 mg/L			↑ [65]			↓ [65]		↑ [65]						
	1-10 dpf	28	7.5 mg/L	1.5 mg/L			↑ [65]			↑ [65]		↑ [65]						
	0-10 dpf	28	7.5 mg/L	1.5 – 1.9 mg/L						↓ [66]								
	0-12 dpf	25	6.5 mg/L	0.8 mg/L						↓ [56]								

Journal of Experimental Biology • Supplementary information

Class and species	Stage	Body Temp (°C)	$[O_2]$ Control	$[O_2]$ Hypoxia	(A) Body mass		(B) Heart mass		(C) Heart-body weight ratio	(D) Heart rate		(E) O_2 carrying capacity	(F) Mito capacity		(G) Cardiac Fibrosis		(H) Sympathetic activity	
**FISH**					Emb	Juv or Adult	Emb	Juv or Adult	Emb	Emb	Juv or Adult	Emb	Emb	Juv or Adult	Emb	Juv or Adult	Emb	Juv or Adult
	0-3 dpf	28	7.5 mg/L	1.5 – 1.9 mg/L						↓ [66]								
	0-30 dpf	28	7.5 mg /L	4.3 mg /L	— [58]	↓ [58]				↑ [58]								
	1-5 dpf	31	7.5 mg/L	3.8 mg/L	— [57]		↑ [57]			↑ [57]								
**Rainbow trout *(Oncorhynchus mykiss)*: 60-90 days**	25-36 dpf	10	10 mg /L	5 mg /L	↓ [67]													
	0-57 dpf	11	100%	34%	↓ [68]	↑ [68]												
	0-45 dpf	10	100%	30%	↓ [69]							↑ [69]						
**Chinook salmon *(Oncorhynchus tshawytsch)*: 90-150 days**	0-1 dph	10	10 mg /L	5.5 mg /L		↓ [70]												
	0-1 dph	15	10 mg /L	5.5 mg /L		↓ [70]												
***Small-spotted catshark* (*Scyliorhinus canicula*): 240-270 days**	0-28 wpf	15 and 20	100% air sat	50% air sat	— [71]													
**Grass carp (*Ctenopharyngodon idellus*): 1-3 days**	0-1 dpf	22	7.0 mg/L	1.0 mg/L	↓ [72]													

Class and species	Stage	Body Temp (°C)	$[O_2]$ Control	$[O_2]$ Hypoxia	(A) Body mass		(B) Heart mass	(C) Heart-body weight ratio		(D) Heart rate		(E) O_2 carrying capacity	(F) Mito capacity		(G) Cardiac Fibrosis		(H) Sympathetic activity	
**FISH**					**Emb**	**Juv or Adult**	**Emb**	**Juv or Adult**	**Emb**	**Emb**	**Juv or Adult**	**Emb**	**Emb**	**Juv or Adult**	**Emb**	**Juv or Adult**	**Emb**	**Juv or Adult**
**European seabass (*Dicentrarchus labrax*): 9 days**	28-50 dph	15	7.35 mg/L	2.95 mg/L	— [73]	↓ [73]												
	28-50 dph	20	7.35 mg/L	2.95 mg/L	— [73]	— [73]												
	30-38 dph	19	9.3 mg/L	3,7 mg/L		↓ [74]												
**Atlantic salmon (*Salmo salar*): 57-75 days**	0-100 dpf	8	11.9 mg/L	5.96 mg/L		— [75]												

Abbreviations: HB, hypobaric hypoxic; dpf, days post fertilisation; dph, days post hatch; wpf, weeks post-fertilisation; di, days of incubation; $\%O_2$, % oxygen saturation. Average gestation period for each species is given for each species in column 1.

Journal of Experimental Biology • Supplementary information

Table S2. Effects of chronic developmental hypoxia on mammalian body mass and cardiovascular parameters

MAMMALS	Stage (GD)	Body Temp (°C)	$[O_2]$ Control	$[O_2]$ Hypoxia	(A) Body mass		(B) Heart mass		(C) Heart-body weight ratio	(D) Heart rate		(E) O_2 carrying capacity	(F) Mito capacity		(G) Cardiac Fibrosis		(H) Sympathetic activity	
					Fetal	Juv or Adult	Fetal	Juv or Adult	Fetal	Fetal	Juv or Adult	Fetal	Fetal	Juv or Adult	Fetal	Juv or Adult	Fetal	Juv or Adult
Rat (*Rattus norvegicus*): 21-23 days	6-20	37	21%O_2	13%O_2	— 76-78	— 77	— 76,77	— 77	— 76,77		— 77	↑ 78	↓ 79<br>— 79					↑ 77
	10-20	37	21%O_2	12%O_2							— 80							↑ 80
	15-20	37	21%O_2	10%O_2	↓ 81,82	— 81	— 82	— 82	— 82		— 81	↑ 82						
	15-21	37	21%O_2	10.5%O_2	↓ 83		— 83		↑ 83						↑ 83			
	15-21	37	21%O_2	12%O_2	↓ 84,85		— 85			— 85					↑ 85			
	15-21	37	21%O_2	11%O_2	↓ 86	— 86	↑ 86		↑ 86							↑ 86		
Mouse (*Mus musculus*): 19-21 days	14-20	37	21%O_2	12%O_2	↓ 87	— 87										↑ 87		
	6-18	37	21%O_2	14%O_2										↑↓ 88				
Guinea pig (*Cavia porcellus*): 59 – 72 days	58-70	38	21%O_2	12%O_2	— 89													
	49-63	38	21%O_2	10.5%O_2	↓ 90		— 90		↑ 90						↑ 90			
	35-60	38		Uterine Artery Constriction	— 91	↓ 91												
	25-64	38	21%O_2	10%O_2	↓ 92								↑↓ 92					
	50-64	38	21%O_2	10.5%O_2	↓ 92								— 93<br>↓ 92	↓ 94,95<br>— 95				

Table S3. Common pathological cardiovascular signatures in juvenile and adult vertebrates that were exposed to chronic developmental hypoxia

Pathological signatures observed in mammals from hypoxic pregnancies	Birds	Crocodilians	Squamates and testudines	Fish
Catch up growth [81,84,96-100]	Yes [4,24] (2/5)	Yes [35-37] (3/5)	Yes [45-47] (3/4)	Yes [68,73,75] (3/9)
Increased heart/body weight ratio [85]	Yes [3,5,11,13,24] (5/10)	Yes [35-39] (4/4)	Yes [46,47] (2/3)	Unknown
Fibrosis [85,101,102]	Yes [15] (1/2)	Unknown	Unknown	Unknown
Ventricular wall thinning [99]	Yes [15] (1/2)	Unknown	Unknown	Unknown
Ventricular wall thickening [103]	Yes [23,104] (1/3)	Unknown	Unknown	Unknown
Aortic wall thickening [105]	Unknown	Unknown	No [106] (1/1)	Unknown
Systemic hypertension [22,80,100,102,105,107]	Yes [23] (1/1)	No [108] (1/1)	No [44,106] (2/2)	Unknown
Enhanced Sympathetic tone [96,98,109]	Yes [24] (1/1)	Yes [35,36] (2/2)	Unknown	Unknown
Mitochondrial dysfunction [88,94,95,107]	Unknown	No [38] (1/1)	No [45,47] (2/2)	Unknown
Increased sensitivity to hypoxia, anoxia or ischemia [80,81,85,98,109-111]	Unknown	No [36,37,108] (3/3)	No [46,112] (2/2)	Unknown
Diastolic dysfunction [85,103,109]	Yes [15] (1/3)	No [36,39] (2/2)	Unknown	Unknown
Systolic dysfunction [107]	Yes [15,24] (2/3)	No [36,39] (2/2)	Unknown	Unknown
Enhanced contractility [96,109]	Yes [23] (1/2)	No [36] (1/1)	Unknown	Unknown
Pulmonary hypertension [103,113,114]	Unknown	Unknown	No [106] (1/1)	Unknown

References in first column are from mammalian studies of chronic developmental hypoxia (CDH). Red and green colours indicate the presence or absence of the response, respectively. Grey colour indicates that the parameter has yet to be studied in this vertebrate class. Fractions in brackets indicate the percentage of papers that found the result (e.g. 1/3 = one in three papers found this result).

1 Dzialowski, E. M., von Plettenberg, D., Elmonoufy, N. A. & Burggren, W. W. Chronic hypoxia alters the physiological and morphological trajectories of developing chicken embryos. *Comparative Biochemistry and Physiology Part A: Molecular & Integrative Physiology* **131**, 713-724 (2002).

2 Chan, T. & Burggren, W. Hypoxic incubation creates differential morphological effects during specific developmental critical windows in the embryo of the chicken (Gallus gallus). *Respir Physiol Neurobiol* **145**, 251-263 (2005). https://doi.org/10.1016/j.resp.2004.09.005

3 Villamor, E. *et al.* Chronic in ovo hypoxia decreases pulmonary arterial contractile reactivity and induces biventricular cardiac enlargement in the chicken embryo. *American Journal of Physiology-Regulatory, Integrative and Comparative Physiology* **287**, R642-R651 (2004).

4 Zoer, B. *et al.* Effects of prenatal hypoxia on pulmonary vascular reactivity in chickens prone to pulmonary hypertension. *J Physiol Pharmacol* **60**, 119-130 (2009).

5 Azzam, M. A. & Mortola, J. P. Organ growth in chicken embryos during hypoxia: implications on organ "sparing" and "catch-up growth". *Respiratory physiology & neurobiology* **159**, 155-162 (2007).

6 Starr, V. J. & Dzialowski, E. M. Developing chicken cardiac muscle mitochondria are resistant to variations in incubation oxygen levels. *Curr Res Physiol* **5**, 151-157 (2022). https://doi.org/10.1016/j.crphys.2022.03.001

7 Ruijtenbeek, K. *et al.* Chronic hypoxia stimulates periarterial sympathetic nerve development in chicken embryo. *Circulation* **102**, 2892-2897 (2000).

8 Dusseau, J. W. & Hutchins, P. M. Hypoxia-induced angiogenesis in chick chorioallantoic membranes: a role for adenosine. *Respiration Physiology* **71**, 33-44 (1988). https://doi.org/https://doi.org/10.1016/0034-5687(88)90113-2

9 Stock, M. K. & Metcalfe, J. Modulation of growth and metabolism of the chick embryo by a brief (72-hr) change in oxygen availability. *The Journal of Experimental zoology. Supplement: Published Under Auspices of the American Society of Zoologists and the Division of Comparative Physiology and Biochemistry* **1**, 351-356 (1987).

10 Lindgren, I. & Altimiras, J. Sensitivity of organ growth to chronically low oxygen levels during incubation in Red Junglefowl and domesticated chicken breeds. *Poult Sci* **90**, 126-135 (2011). https://doi.org/10.3382/ps.2010-00996

11 Miller, S. L., Green, L. R., Peebles, D. M., Hanson, M. A. & Blanco, C. E. Effects of chronic hypoxia and protein malnutrition on growth in the developing chick. *Am J Obstet Gynecol* **186**, 261-267 (2002). https://doi.org/10.1067/mob.2002.119629

12 Rouwet, E. V. *et al.* Hypoxia induces aortic hypertrophic growth, left ventricular dysfunction, and sympathetic hyperinnervation of peripheral arteries in the chick embryo. *Circulation* **105**, 2791-2796 (2002). https://doi.org/10.1161/01.cir.0000017497.47084.06

13 Salinas, C. *et al.* Cardiac and vascular disease prior to hatching in chick embryos incubated at high altitude. *Journal of Developmental Origins of Health and Disease* **1**, 60-66 (2010).

14 Sharma, S. K. *et al.* Impact of hypoxia on early chick embryo growth and cardiovascular function. *Pediatric research* **59**, 116-120 (2006).

15 Tintu, A. *et al.* Hypoxia induces dilated cardiomyopathy in the chick embryo: mechanism, intervention, and long-term consequences. *PLoS One* **4**, e5155 (2009). https://doi.org/10.1371/journal.pone.0005155

16 Jonker, S. S., Giraud, G. D., Espinoza, H. M., Davis, E. N. & Crossley 2nd, D. A. Effects of chronic hypoxia on cardiac function measured by pressure-volume catheter in fetal chickens. *American Journal of Physiology-Regulatory, Integrative and Comparative Physiology* **308**, R680-R689 (2015).

17 Itani, N. *et al.* Protective effects of pravastatin on the embryonic cardiovascular system during hypoxic development. *The FASEB Journal* **34**, 16504-16515 (2020). https://doi.org/https://doi.org/10.1096/fj.202001743R
18 Itani, N., Skeffington, K. L., Beck, C., Niu, Y. & Giussani, D. A. Melatonin rescues cardiovascular dysfunction during hypoxic development in the chick embryo. *Journal of pineal research* **60**, 16-26 (2016).
19 Salinas, C. E., Villena, M., Blanco, C. E. & Giussani, D. A. Adrenocortical suppression in highland chick embryos is restored during incubation at sea level. *High Altitude Medicine & Biology* **12**, 79-87 (2011).
20 Giussani, D. A., Salinas, C. E., Villena, M. & Blanco, C. E. The role of oxygen in prenatal growth: studies in the chick embryo. *The Journal of Physiology* **585**, 911-917 (2007). https://doi.org/https://doi.org/10.1113/jphysiol.2007.141572
21 Itani, N., Skeffington, K. L., Beck, C. & Giussani, D. A. Sildenafil therapy for fetal cardiovascular dysfunction during hypoxic development: studies in the chick embryo. *The Journal of physiology* **595**, 1563-1573 (2017).
22 Botting, K. J. *et al.* Translatable mitochondria-targeted protection against programmed cardiovascular dysfunction. *Sci Adv* **6**, eabb1929 (2020). https://doi.org/10.1126/sciadv.abb1929
23 Skeffington, K. L. *et al.* Hypertension Programmed in Adult Hens by Isolated Effects of Developmental Hypoxia In Ovo. *Hypertension* **76**, 533-544 (2020). https://doi.org/10.1161/hypertensionaha.120.15045
24 Lindgren, I. & Altimiras, J. Chronic prenatal hypoxia sensitizes β-adrenoceptors in the embryonic heart but causes postnatal desensitization. *American Journal of Physiology-Regulatory, Integrative and Comparative Physiology* **297**, R258-R264 (2009).
25 Lindgren, I. & Altimiras, J. Prenatal hypoxia programs changes in β-adrenergic signaling and postnatal cardiac contractile dysfunction. *American Journal of Physiology-Regulatory, Integrative and Comparative Physiology* **305**, R1093-R1101 (2013).
26 Lindgren, I., Dane Crossley, I., Villamor, E. & Altimiras, J. Hypotension in the chronically hypoxic chicken embryo is related to the β-adrenergic response of chorioallantoic and femoral arteries and not to bradycardia. *American Journal of Physiology-Regulatory, Integrative and Comparative Physiology* **301**, R1161-R1168 (2011). https://doi.org/10.1152/ajpregu.00458.2010
27 Lourens, A., van den Brand, H., Heetkamp, M. J. W., Meijerhof, R. & Kemp, B. Effects of Eggshell Temperature and Oxygen Concentration on Embryo Growth and Metabolism During Incubation. *Poultry Science* **86**, 2194-2199 (2007). https://doi.org/https://doi.org/10.1093/ps/86.10.2194
28 Snyder, G. K., Black, C. P. & Birchard, G. F. Development and metabolism during hypoxia in embryos of high altitude Anser indicus versus sea level Branta canadensis geese. *Physiological Zoology* **55**, 113-123 (1982).
29 Snyder, G. K., Byers, R. L. & Kayar, S. R. Effects of hypoxia on tissue capillarity in geese. *Respiration physiology* **58**, 151-160 (1984).
30 Crossley, D. A. & Altimiras, J. Cardiovascular development in embryos of the American alligator Alligator mississippiensis: effects of chronic and acute hypoxia. *Journal of Experimental Biology* **208**, 31-39 (2005).
31 Eme, J., Crossley, D. A., 2nd & Hicks, J. W. Role of the left aortic arch and blood flows in embryonic American alligator (Alligator mississippiensis). *Journal of comparative physiology. B, Biochemical, systemic, and environmental physiology* **181**, 391-401 (2011). https://doi.org/10.1007/s00360-010-0494-6
32 Owerkowicz, T., Elsey, R. M. & Hicks, J. W. Atmospheric oxygen level affects growth trajectory, cardiopulmonary allometry and metabolic rate in the American alligator (Alligator mississippiensis). *Journal of Experimental Biology* **212**, 1237-1247 (2009). https://doi.org/10.1242/jeb.023945

Journal of Experimental Biology • Supplementary information

33 Tate, K. B. *et al.* Periods of cardiovascular susceptibility to hypoxia in embryonic american alligators (Alligator mississippiensis). *American journal of physiology. Regulatory, integrative and comparative physiology* **310**, R1267-R1278 (2016). https://doi.org/10.1152/ajpregu.00320.2015

34 Eme, J., Altimiras, J., Hicks, J. W. & Crossley, D. A. Hypoxic alligator embryos: Chronic hypoxia, catecholamine levels and autonomic responses of in ovo alligators. *Comparative Biochemistry and Physiology Part A: Molecular & Integrative Physiology* **160**, 412-420 (2011). https://doi.org/https://doi.org/10.1016/j.cbpa.2011.07.010

35 Joyce, W., Miller, T. E., Elsey, R. M., Wang, T. & Crossley, D. A., 2nd. The effects of embryonic hypoxic programming on cardiovascular function and autonomic regulation in the American alligator (Alligator mississippiensis) at rest and during swimming. *J Comp Physiol B* **188**, 967-976 (2018). https://doi.org/10.1007/s00360-018-1181-2

36 Smith, B., Crossley, J. L., Elsey, R. M., Hicks, J. W. & Crossley, D. A., 2nd. Embryonic developmental oxygen preconditions cardiovascular functional response to acute hypoxic exposure and maximal β-adrenergic stimulation of anesthetized juvenile American alligators (Alligator mississippiensis). *J Exp Biol* **222** (2019). https://doi.org/10.1242/jeb.205419

37 Crossley, J. L. *et al.* Developmental oxygen preadapts ventricular function of juvenile American alligators, Alligator mississippiensis. *American Journal of Physiology-Regulatory, Integrative and Comparative Physiology* **323**, R739-R748 (2022). https://doi.org/10.1152/ajpregu.00059.2022

38 Galli, G. L. *et al.* Developmental plasticity of mitochondrial function in American alligators, Alligator mississippiensis. *Am J Physiol Regul Integr Comp Physiol* **311**, R1164-r1172 (2016). https://doi.org/10.1152/ajpregu.00107.2016

39 Smith, B. *et al.* Exposure to hypoxia during embryonic development affects blood flow patterns and heart rate in juvenile American alligators during digestion. *Comparative Biochemistry and Physiology Part A: Molecular & Integrative Physiology* **282**, 111440 (2023). https://doi.org/https://doi.org/10.1016/j.cbpa.2023.111440

40 Warburton, S. J., Hastings, D. & Wang, T. Responses to chronic hypoxia in embryonic alligators. *J Exp Zool* **273**, 44-50 (1995). https://doi.org/10.1002/jez.1402730106

41 Eme, J., Rhen, T. & Crossley, D. A., 2nd. Adjustments in cholinergic, adrenergic and purinergic control of cardiovascular function in snapping turtle embryos (Chelydra serpentina) incubated in chronic hypoxia. *J Comp Physiol B* **184**, 891-902 (2014). https://doi.org/10.1007/s00360-014-0848-6

42 Eme, J. *et al.* Plasticity of cardiovascular function in snapping turtle embryos (Chelydra serpentina): chronic hypoxia alters autonomic regulation and gene expression. *Am J Physiol Regul Integr Comp Physiol* **304**, R966-979 (2013). https://doi.org/10.1152/ajpregu.00595.2012

43 Eme, J., Tate, K. B., Rhen, T. & Crossley, D. A., 2nd. Cardiovascular responses to putative chemoreceptor stimulation of embryonic common snapping turtles (Chelydra serpentina) chronically incubated in hypoxia (10% O(2)). *Comp Biochem Physiol A Mol Integr Physiol* **259**, 110977 (2021). https://doi.org/10.1016/j.cbpa.2021.110977

44 Wearing, O. H., Eme, J., Rhen, T. & Crossley, D. A., 2nd. Phenotypic plasticity in the common snapping turtle (Chelydra serpentina): long-term physiological effects of chronic hypoxia during embryonic development. *Am J Physiol Regul Integr Comp Physiol* **310**, R176-184 (2016). https://doi.org/10.1152/ajpregu.00293.2015

45 Ruhr, I. M. *et al.* Developmental plasticity of cardiac anoxia-tolerance in juvenile common snapping turtles (<i>Chelydra serpentina</i>). *Proceedings of the Royal Society B: Biological Sciences* **286**, 20191072 (2019). https://doi.org/doi:10.1098/rspb.2019.1072

46 Ruhr, I. *et al.* Developmental programming of DNA methylation and gene expression patterns is associated with extreme cardiovascular tolerance to anoxia in the common snapping turtle. *Epigenetics & Chromatin* **14**, 42 (2021). https://doi.org/10.1186/s13072-021-00414-7
47 Galli, G. L. J., Ruhr, I. M., Crossley, J. & Crossley, D. A. The Long-Term Effects of Developmental Hypoxia on Cardiac Mitochondrial Function in Snapping Turtles. *Frontiers in Physiology* **12** (2021). https://doi.org/10.3389/fphys.2021.689684
48 Tate, K. B., Kohl, Z. F., Eme, J., Rhen, T. & Crossley, D. A., 2nd. Critical Windows of Cardiovascular Susceptibility to Developmental Hypoxia in Common Snapping Turtle (Chelydra serpentina) Embryos. *Physiol Biochem Zool* **88**, 103-115 (2015). https://doi.org/10.1086/677683
49 Kam, Y. C. Physiological effects of hypoxia on metabolism and growth of turtle embryos. *Respir Physiol* **92**, 127-138 (1993). https://doi.org/10.1016/0034-5687(93)90033-7
50 Parker, S. L. & Dimkovikj, V. H. Effects of regional hypoxia and incubation temperature on growth, differentiation, heart mass, and oxygen consumption in embryos of the leopard gecko (Eublepharis macularius). *Comp Biochem Physiol A Mol Integr Physiol* **227**, 51-59 (2019). https://doi.org/10.1016/j.cbpa.2018.09.006
51 Tang, W., Zhao, B., Chen, Y. & Du, W. Reduced egg shell permeability affects embryonic development and hatchling traits in Lycodon rufozonatum and Pelodiscus sinensis. *Integr Zool* **13**, 58-69 (2018). https://doi.org/10.1111/1749-4877.12269
52 Cordero, G. A. *et al.* Physiological plasticity in lizard embryos exposed to high-altitude hypoxia. *Journal of Experimental Zoology Part A: Ecological and Integrative Physiology* **327**, 423-432 (2017). https://doi.org/https://doi.org/10.1002/jez.2115
53 Kouyoumdjian, L. *et al.* Transplanting gravid lizards to high elevation alters maternal and embryonic oxygen physiology, but not reproductive success or hatchling phenotype. *Journal of Experimental Biology* **222** (2019). https://doi.org/10.1242/jeb.206839
54 Souchet, J. *et al.* High temperatures limit developmental resilience to high-elevation hypoxia in the snake Natrix maura (Squamata: Colubridae). *Biological Journal of the Linnean Society* **132**, 116-133 (2020). https://doi.org/10.1093/biolinnean/blaa182
55 Souchet, J. *et al.* High-elevation hypoxia impacts perinatal physiology and performance in a potential montane colonizer. *Integrative Zoology* **15**, 544-557 (2020). https://doi.org/https://doi.org/10.1111/1749-4877.12468
56 Bagatto, B. Ontogeny of cardiovascular control in zebrafish (Danio rerio): effects of developmental environment. *Comp Biochem Physiol A Mol Integr Physiol* **141**, 391-400 (2005). https://doi.org/10.1016/j.cbpb.2005.07.002
57 Jacob, E., Drexel, M., Schwerte, T. & Pelster, B. Influence of hypoxia and of hypoxemia on the development of cardiac activity in zebrafish larvae. *American Journal of Physiology-Regulatory, Integrative and Comparative Physiology* **283**, R911-R917 (2002).
58 Barrionuevo, W. R., Fernandes, M. N. & Rocha, O. Aerobic and anaerobic metabolism for the zebrafish, Danio rerio, reared under normoxic and hypoxic conditions and exposed to acute hypoxia during development. *Braz J Biol* **70**, 425-434 (2010). https://doi.org/10.1590/s1519-69842010000200027
59 Kajimura, S., Aida, K. & Duan, C. Insulin-like growth factor-binding protein-1 (IGFBP-1) mediates hypoxia-induced embryonic growth and developmental retardation. *Proc Natl Acad Sci U S A* **102**, 1240-1245 (2005). https://doi.org/10.1073/pnas.0407443102
60 Ton, C., Stamatiou, D. & Liew, C.-C. Gene expression profile of zebrafish exposed to hypoxia during development. *Physiological Genomics* **13**, 97-106 (2003).

Journal of Experimental Biology • Supplementary information

61 Cypher, A. D., Fetterman, B. & Bagatto, B. Vascular parameters continue to decrease post-exposure with simultaneous, but not individual exposure to BPA and hypoxia in zebrafish larvae. *Comp Biochem Physiol C Toxicol Pharmacol* **206-207**, 11-16 (2018). https://doi.org/10.1016/j.cbpc.2018.02.002
62 Moore, F. B., Hosey, M. & Bagatto, B. Cardiovascular system in larval zebrafish responds to developmental hypoxia in a family specific manner. *Front Zool* **3**, 4 (2006). https://doi.org/10.1186/1742-9994-3-4
63 Schwerte, T., Überbacher, D. & Pelster, B. Non-invasive imaging of blood cell concentration and blood distribution in hypoxic incubated zebrafish in vivo (Danio rerio). *J. Exp. Biol* **206**, 1299-1307 (2003).
64 Steele, S. L., Ekker, M. & Perry, S. F. Interactive effects of development and hypoxia on catecholamine synthesis and cardiac function in zebrafish (Danio rerio). *Journal of Comparative Physiology B* **181**, 527-538 (2011).
65 Yaqoob, N. & Schwerte, T. Cardiovascular and respiratory developmental plasticity under oxygen depleted environment and in genetically hypoxic zebrafish (Danio rerio). *Comparative Biochemistry and Physiology Part A: Molecular & Integrative Physiology* **156**, 475-484 (2010).
66 Steele, S. L. *et al.* Loss of M2 muscarinic receptor function inhibits development of hypoxic bradycardia and alters cardiac β-adrenergic sensitivity in larval zebrafish (Danio rerio). *American Journal of Physiology-Regulatory, Integrative and Comparative Physiology* **297**, R412-R420 (2009).
67 Ciuhandu, C. S., Stevens, E. D. & Wright, P. A. The effect of oxygen on the growth of Oncorhynchus mykiss embryos with and without a chorion. *Journal of Fish Biology* **67**, 1544-1551 (2005). https://doi.org/https://doi.org/10.1111/j.1095-8649.2005.00856.x
68 Johnston, E. F., Alderman, S. L. & Gillis, T. E. Chronic hypoxia exposure of trout embryos alters swimming performance and cardiac gene expression in larvae. *Physiological and Biochemical Zoology* **86**, 567-575 (2013).
69 Bianchini, K. & Wright, P. A. Hypoxia delays hematopoiesis: retention of embryonic hemoglobin and erythrocytes in larval rainbow trout, Oncorhynchus mykiss, during chronic hypoxia exposure. *Journal of Experimental Biology* **216**, 4415-4425 (2013). https://doi.org/10.1242/jeb.083337
70 Del Rio, A. M., Davis, B. E., Fangue, N. A. & Todgham, A. E. Combined effects of warming and hypoxia on early life stage Chinook salmon physiology and development. *Conservation Physiology* **7** (2019). https://doi.org/10.1093/conphys/coy078
71 Musa, S. M., Ripley, D. M., Moritz, T. & Shiels, H. A. Ocean warming and hypoxia affect embryonic growth, fitness and survival of small-spotted catsharks, Scyliorhinus canicula. *Journal of Fish Biology* **97**, 257-264 (2020). https://doi.org/https://doi.org/10.1111/jfb.14370
72 Sun, C.-F., Tao, Y., Jiang, X.-Y. & Zou, S.-M. IGF binding protein 1 is correlated with hypoxia-induced growth reduce and developmental defects in grass carp (Ctenopharyngodon idellus) embryos. *General and Comparative Endocrinology* **172**, 409-415 (2011). https://doi.org/https://doi.org/10.1016/j.ygcen.2011.04.005
73 Cadiz, L. *et al.* Early exposure to chronic hypoxia induces short- and long-term regulation of hemoglobin gene expression in European sea bass (Dicentrarchus labrax). *J Exp Biol* **220**, 3119-3126 (2017). https://doi.org/10.1242/jeb.160713
74 Vanderplancke, G. *et al.* Hypoxic episode during the larval period has long-term effects on European sea bass juveniles (Dicentrarchus labrax). *Marine biology* **162**, 367-376 (2015).
75 Wood, A. T., Clark, T. D., Andrewartha, S. J., Elliott, N. G. & Frappell, P. B. Developmental hypoxia has negligible effects on long-term hypoxia tolerance and aerobic metabolism of Atlantic salmon (Salmo salar). *Physiological and Biochemical Zoology* **90**, 494-501 (2017).

Journal of Experimental Biology • Supplementary information

76 Herrera, E. A. *et al.* Morphological and Functional Alterations in the Aorta of the Chronically Hypoxic Fetal Rat. *Journal of Vascular Research* **49**, 50-58 (2012). https://doi.org/10.1159/000330666

77 Giussani, D. A. *et al.* Developmental Programming of Cardiovascular Dysfunction by Prenatal Hypoxia and Oxidative Stress. *PLOS ONE* **7**, e31017 (2012). https://doi.org/10.1371/journal.pone.0031017

78 Nuzzo, A. M. *et al.* Placental Adaptation to Early-Onset Hypoxic Pregnancy and Mitochondria-Targeted Antioxidant Therapy in a Rodent Model. *Am J Pathol* **188**, 2704-2716 (2018). https://doi.org/10.1016/j.ajpath.2018.07.027

79 Smith, K. M. *et al.* Chronic developmental hypoxia alters mitochondrial oxidative capacity and reactive oxygen species production in the fetal rat heart in a sex-dependent manner. *Journal of Pineal Research* **73**, e12821 (2022).

80 Rook, W., Johnson, C. D., Coney, A. M. & Marshall, J. M. Prenatal hypoxia leads to increased muscle sympathetic nerve activity, sympathetic hyperinnervation, premature blunting of neuropeptide Y signaling, and hypertension in adult life. *Hypertension* **64**, 1321-1327 (2014). https://doi.org/10.1161/hypertensionaha.114.04374

81 Xue, Q. & Zhang, L. Prenatal hypoxia causes a sex-dependent increase in heart susceptibility to ischemia and reperfusion injury in adult male offspring: role of protein kinase C epsilon. *J Pharmacol Exp Ther* **330**, 624-632 (2009). https://doi.org/10.1124/jpet.109.153239

82 Hansell, J. A. *et al.* Maternal melatonin: Effective intervention against developmental programming of cardiovascular dysfunction in adult offspring of complicated pregnancy. *Journal of Pineal Research* **72**, e12766 (2022). https://doi.org/https://doi.org/10.1111/jpi.12766

83 Tong, W., Xue, Q., Li, Y. & Zhang, L. Maternal hypoxia alters matrix metalloproteinase expression patterns and causes cardiac remodeling in fetal and neonatal rats. *Am J Physiol Heart Circ Physiol* **301**, H2113-2121 (2011). https://doi.org/10.1152/ajpheart.00356.2011

84 Morton, J. S., Rueda-Clausen, C. F. & Davidge, S. T. Mechanisms of endothelium-dependent vasodilation in male and female, young and aged offspring born growth restricted. *Am J Physiol Regul Integr Comp Physiol* **298**, R930-938 (2010). https://doi.org/10.1152/ajpregu.00641.2009

85 Xu, Y., Williams, S. J., O'Brien, D. & Davidge, S. T. Hypoxia or nutrient restriction during pregnancy in rats leads to progressive cardiac remodeling and impairs postischemic recovery in adult male offspring. *Faseb j* **20**, 1251-1253 (2006). https://doi.org/10.1096/fj.05-4917fje

86 Rueda-Clausen, C. F. *et al.* Effects of hypoxia-induced intrauterine growth restriction on cardiac siderosis and oxidative stress. *Journal of Developmental Origins of Health and Disease* **3**, 350-357 (2012). https://doi.org/10.1017/S2040174412000219

87 Walton, S. L., Singh, R. R., Tan, T., Paravicini, T. M. & Moritz, K. M. Late gestational hypoxia and a postnatal high salt diet programs endothelial dysfunction and arterial stiffness in adult mouse offspring. *The Journal of Physiology* **594**, 1451-1463 (2016). https://doi.org/https://doi.org/10.1113/JP271067

88 Hellgren, K. T., Premanandhan, H., Quinn, C. J., Trafford, A. W. & Galli, G. L. J. Sex-dependent effects of developmental hypoxia on cardiac mitochondria from adult murine offspring. *Free Radic Biol Med* **162**, 490-499 (2021). https://doi.org/10.1016/j.freeradbiomed.2020.11.004

89 Thompson, L. P. & Weiner, C. P. Effects of acute and chronic hypoxia on nitric oxide–mediated relaxation of fetal guinea pig arteries. *American Journal of Obstetrics and Gynecology* **181**, 105-111 (1999). https://doi.org/https://doi.org/10.1016/S0002-9378(99)70444-8

90 Evans, L. C., Liu, H., Pinkas, G. A. & Thompson, L. P. Chronic hypoxia increases peroxynitrite, MMP9 expression, and collagen accumulation in fetal guinea pig hearts. *Pediatr Res* **71**, 25-31 (2012). https://doi.org/10.1038/pr.2011.10

91 Krause, B. J. *et al.* Adult vascular dysfunction in foetal growth-restricted guinea-pigs is associated with a neonate-adult switching in Nos3 DNA methylation. *Acta Physiologica* **227**, e13328 (2019). https://doi.org/https://doi.org/10.1111/apha.13328

92 Song, H., Polster, B. M. & Thompson, L. P. Chronic hypoxia alters cardiac mitochondrial complex protein expression and activity in fetal guinea pigs in a sex-selective manner. *American Journal of Physiology-Regulatory, Integrative and Comparative Physiology* (2021). https://doi.org/10.1152/ajpregu.00004.2021

93 Al-Hasan, Y. M. *et al.* Chronic hypoxia impairs cytochrome oxidase activity via oxidative stress in selected fetal Guinea pig organs. *Reprod Sci* **20**, 299-307 (2013). https://doi.org/10.1177/1933719112453509

94 Al-Hasan, Y. M., Pinkas, G. A. & Thompson, L. P. Prenatal Hypoxia Reduces Mitochondrial Protein Levels and Cytochrome c Oxidase Activity in Offspring Guinea Pig Hearts. *Reprod Sci* **21**, 883-891 (2014). https://doi.org/10.1177/1933719113518981

95 Thompson, L. P., Song, H. & Polster, B. M. Fetal Programming and Sexual Dimorphism of Mitochondrial Protein Expression and Activity of Hearts of Prenatally Hypoxic Guinea Pig Offspring. *Oxid Med Cell Longev* **2019**, 7210249 (2019). https://doi.org/10.1155/2019/7210249

96 Giussani, D. A. *et al.* Developmental programming of cardiovascular dysfunction by prenatal hypoxia and oxidative stress. *PloS one* **7**, e31017 (2012).

97 Li, G., Bae, S. & Zhang, L. Effect of prenatal hypoxia on heat stress-mediated cardioprotection in adult rat heart. *Am J Physiol Heart Circ Physiol* **286**, H1712-1719 (2004). https://doi.org/10.1152/ajpheart.00898.2003

98 Li, G. *et al.* Effect of fetal hypoxia on heart susceptibility to ischemia and reperfusion injury in the adult rat. *J Soc Gynecol Investig* **10**, 265-274 (2003). https://doi.org/10.1016/s1071-5576(03)00074-1

99 Hansell, J. A. *et al.* Maternal melatonin: Effective intervention against developmental programming of cardiovascular dysfunction in adult offspring of complicated pregnancy. *J Pineal Res* **72**, e12766 (2022). https://doi.org/10.1111/jpi.12766

100 Brain, K. L. *et al.* Intervention against hypertension in the next generation programmed by developmental hypoxia. *PLoS Biol* **17**, e2006552 (2019). https://doi.org/10.1371/journal.pbio.2006552

101 Wang, L., Li, M., Huang, Z. & Wang, Z. The influence of hypoxia during different pregnancy stages on cardiac collagen accumulation in the adult offspring. *Biomed Res Int* **2014**, 419805 (2014). https://doi.org/10.1155/2014/419805

102 Walton, S. L. *et al.* Prenatal hypoxia leads to hypertension, renal renin-angiotensin system activation and exacerbates salt-induced pathology in a sex-specific manner. *Scientific reports* **7**, 8241-8241 (2017). https://doi.org/10.1038/s41598-017-08365-4

103 Rueda-Clausen, C. F., Morton, J. S. & Davidge, S. T. Effects of hypoxia-induced intrauterine growth restriction on cardiopulmonary structure and function during adulthood. *Cardiovascular Research* **81**, 713-722 (2008). https://doi.org/10.1093/cvr/cvn341

104 Salinas, C. E., Blanco, C. E., Villena, M. & Giussani, D. A. High-altitude hypoxia and echocardiographic indices of pulmonary hypertension in male and female chickens at adulthood. *Circ J* **78**, 1459-1464 (2014). https://doi.org/10.1253/circj.cj-13-1329

105 Zanardo, V. *et al.* Fetal aortic wall thickness: a marker of hypertension in IUGR children? *Hypertens Res* **36**, 440-443 (2013). https://doi.org/10.1038/hr.2012.219

106 Filogonio, R., Dubansky, B. D., Dubansky, B. H., Leite, C. A. C. & Crossley, D. A., 2nd. Prenatal hypoxia affects scaling of blood pressure and arterial wall mechanics in the common snapping turtle, Chelydra serpentina. *Comp Biochem Physiol A Mol Integr Physiol* **260**, 111023 (2021). https://doi.org/10.1016/j.cbpa.2021.111023
107 Thompson, L. P., Chen, L., Polster, B. M., Pinkas, G. & Song, H. Prenatal hypoxia impairs cardiac mitochondrial and ventricular function in guinea pig offspring in a sex-related manner. *Am J Physiol Regul Integr Comp Physiol* **315**, R1232-R1241 (2018). https://doi.org/10.1152/ajpregu.00224.2018
108 Crossley, J. L. *et al.* Hypoxic incubation at 50% of atmospheric levels shifts the cardiovascular response to acute hypoxia in American alligators, Alligator mississippiensis. *J Comp Physiol B* **193**, 545-556 (2023). https://doi.org/10.1007/s00360-023-01510-8
109 Niu, Y. *et al.* Maternal Allopurinol Prevents Cardiac Dysfunction in Adult Male Offspring Programmed by Chronic Hypoxia During Pregnancy. *Hypertension* **72**, 971-978 (2018). https://doi.org/doi:10.1161/HYPERTENSIONAHA.118.11363
110 Rueda-Clausen, C. F., Morton, J. S., Dolinsky, V. W., Dyck, J. R. & Davidge, S. T. Synergistic effects of prenatal hypoxia and postnatal high-fat diet in the development of cardiovascular pathology in young rats. *Am J Physiol Regul Integr Comp Physiol* **303**, R418-426 (2012). https://doi.org/10.1152/ajpregu.00148.2012
111 Rueda-Clausen, C. F., Morton, J. S., Lopaschuk, G. D. & Davidge, S. T. Long-term effects of intrauterine growth restriction on cardiac metabolism and susceptibility to ischaemia/reperfusion. *Cardiovasc Res* **90**, 285-294 (2011). https://doi.org/10.1093/cvr/cvq363
112 Ruhr, I. M. *et al.* Developmental plasticity of cardiac anoxia-tolerance in juvenile common snapping turtles (Chelydra serpentina). *Proceedings of the Royal Society B: Biological Sciences* **286**, 20191072 (2019). https://doi.org/doi:10.1098/rspb.2019.1072
113 Ding, H. *et al.* Hypoxia in utero increases the risk of pulmonary hypertension in rat offspring and is associated with vasopressin type-2 receptor upregulation. *Mol Med Rep* **22**, 4173-4182 (2020). https://doi.org/10.3892/mmr.2020.11533
114 Li, H. *et al.* Antenatal Hypoxia Affects Pulmonary Artery Contractile Functions via Downregulating L-type Ca(2+) Channels Subunit Alpha1 C in Adult Male Offspring. *J Am Heart Assoc* **10**, e019922 (2021). https://doi.org/10.1161/jaha.120.019922

Journal of Experimental Biology • Supplementary information
